# Factors associated with failure of passive transfer of immunity and morbidity in spring-born beef and dairy calves during the first 30 days of life

**DOI:** 10.3389/fvets.2025.1658532

**Published:** 2026-01-06

**Authors:** Rischi Robinson Male Here, Mark McGee, Catherine McAloon, Andrew W. Byrne, Bernadette Earley

**Affiliations:** 1 Animal and Grassland Research and Innovation Centre, Teagasc, Grange, Dunsany, Co. Meath, Ireland; 2 School of Veterinary Medicine, University College Dublin (UCD), Dublin, Ireland; 3 One Health Scientific Support Unit, Ruminant Animal Health Policy, Department of Agriculture, Food and the Marine (DAFM), Agriculture House, Dublin, Ireland

**Keywords:** calf, immunity tests, immunity cut-offs, calf management, disease susceptibility, risk factors, passive immunity

## Abstract

The failure of passive transfer of immunity (FPT) in calves is associated with increased susceptibility to infectious diseases, leading to higher morbidity rates and reduced herd productivity. The present study evaluated dam and calf characteristics, and herd-level and calf-level management practices associated with passive immune measures, FPT, and morbidity during the first 30 days of life in Irish (suckler) beef and dairy calves. Datasets were collected from herd-level [66 beef farms (391 calves), 77 dairy farms (674 calves)], and calf-level [9 beef farms (377 calves), 8 dairy farms (916 calves)] studies conducted in spring 2015 and 2016, respectively. Passive immunity was assessed in calf serum collected 1–14 days post-birth using ELISA (total IgG; ELISA-IgG), clinical analyzer (total protein; TP-CA), and BRIX refractometer (total solids; TS-BRIX). Calf FPT status was defined using current-cut-offs specific to Irish calves and literature-cut-offs. The current-cut-offs of ELISA-IgG were ≤9 and ≤12 mg/mL for beef and dairy calves, respectively, whereas TP-CA ≤60 g/L and TS-BRIX ≤8.4% were used for both calf types. The literature-cut-offs for both calf types were ELISA-IgG <10 mg/mL, TP-CA <52 g/L, and TS-BRIX <8.4%. Calf morbidity data were recorded by farmers. The datasets were analyzed using mixed-effects linear and logistic regressions, and Cox proportional hazard models. Risk factors associated with passive immune measures (continuous outcome) and/or FPT in beef calves included dam diarrhea vaccination, dam parity, timing of birth in calving season, breed, perinatal problems, and timing and method of colostrum feeding, and in dairy calves included dry period length, dam parity, calf sex, breed, calving supervision, and type of colostrum feeding. Passive immune measures were not associated with morbidity in beef or dairy calves; however, depending on test and cut-offs, FPT was associated with increased morbidity hazard in beef but not in dairy calves. Risk factors for morbidity in beef calves included cow-calf housing location and dam parity, and in dairy calves included cow-calf separation, pen cleaning, dam parity, breed, calving assistance, and colostrum feeding method. The present study underscores the importance of selecting appropriate passive immune tests and cut-offs for FPT and highlights modifiable management practices to improve calf health.

## 1 Introduction

Calves are born with a naïve immune system due placental anatomy, which prevents the maternal transfer of immunoglobulin (Ig; specifically IgG) during gestation (1). Therefore, they depend on timely colostrum intake for adequate immunity transfer. Delayed colostrum feeding post-birth reduces the efficiency of passive transfer and IgG absorption (2), thereby increasing the risk of failure of passive transfer of immunity (FPT). A meta-analysis study reported that calves with FPT are more susceptible to disease or morbidity events, including bovine respiratory disease (BRD) and diarrhea, as well as increased mortality (3). Economically, FPT is estimated to cost €80 and €60 per beef and dairy calf, respectively, which can increase by up to 65% per calf in herds with high FPT prevalence (3).

Determining FPT in calves requires measuring serum or plasma IgG concentrations using direct methods such as radial immunodiffusion (RID), turbidimetric immunoassay, or enzyme linked immunosorbent assay (ELISA) (4). Indirect methods used in laboratory or on-farm settings to estimate IgG concentrations include, measuring serum total protein (TP; using either clinical analyzer or digital refractometer), total solids (using BRIX refractometer), globulin, zinc sulfate turbidity (ZST), or gamma-glutamyl transferase (GGT) (4, 5), with TP and BRIX total solids being most commonly used in research studies. The definition of FPT is based on specific cut-off values for serum IgG concentrations, or equivalents in TP and total solids, which are associated with morbidity or mortality (5–8). Commonly used cut-off values for FPT in beef and dairy calves include serum IgG <10 mg/mL, TP <52 g/L, and BRIX total solids <8.4% (Table 1).

**Table 1:** Prevalence of failure of passive transfer of immunity (FPT) determined using various testing methods and cut-off values in beef and dairy calves internationally.

References	Year of publication	Calf age (day)	Number of calves	Testing method^1^	FPT cut-off	Prevalence (%)	Country
Beef							
Gamsjäger et al. (14)	2023	1–7	420	RID	<10 g/L <24 g/L	5 18	Canada
Perrot et al. (75)	2023	1–9	964	TP	<5.1 g/dL	12.5	France
				BRIX	<8.1%	12.8	
Sustronck et al. (76)	2022	2–3	202	RID	<10 g/L	34.5	Belgium
Bragg et al. (12)	2020	1–13	1,131	RID	<10 g/L <24 g/L	15	Great Britain
						37	
Todd et al. (5)	2018	1–21	1,392	ELISA	≤9 mg/mL	31.3	Ireland
				ZST	≤14 units	40.3	
				TP^2^	≤60 g/L	50.4	
				TP	≤5.8 g/dL	43.8	
				BRIX	≤8.4%	39.2	
				Globulin	≤32 g/L	48.3	
O’Shaughnessy et al. (77)	2015	2–14	82	ZST	<20 units	22	Ireland
Waldner and Rosengren (11)	2009	2–8	601	RID	<8 g/L	5.8	Canada
Dewell et al. (7)	2006	1–3	1,559	RID	<8 g/L	14.2	United States
Filteau et al. (78)	2003	1–7	225	RID	<10 g/L	19	Canada
Dairy							
Michelsen et al. (40)	2025	2–9	834	ELISA	<10 mg/mL	29	Denmark
				BRIX	<8.1%	35.1	
Abdallah et al. (63)	2024	Veal calves^3^	1,084	BRIX	<8.4%	62.7	Canada
Pereira et al. (64)	2024	Dairy-beef calves^3^	1,055	RID	<10 g/L	14.5	United States
				TP	<5.1 g/dL	32.1	
Lichtmannsperger et al. (79)	2023	3–6	250	BRIX	<8.4%	37.2	Austria
Sutter et al. (65)	2023	2–7	3,434	BRIX	<8.1%	4.8	Germany
Denholm et al. (36)	2022	1–7	367	RID	<10 g/L	14.2	Scotland
				ZST	<20 units	46.5	
				BRIX	<8.4%	40.5	
				TP	<5.2 g/dL	29.5	
Roadknight et al. (17)	2022	Bobby calves^3^	3,608	TP	≤52 g/L	36.4	Australia
					<51 g/L	31.1	
Haggerty et al. (80)	2021	1–7	370	RID	<10 g/L	14.1	Scotland
Renaud et al. (16)	2020	1–7	1778	TP	<5.2 g/dL	21.1	Canada
Johnsen et al. (81)	2019	1–2	156	RID	<10 g/L	30.8	Norway and Sweden
Abuelo et al. (82)	2019	1–7	253	RID	<10 g/L	41.9	Australia
Staněk et al. (83)	2019	1–6	1,175	RID	<10 g/L	34.6	Czech Republic
Barry et al. (9)	2019	1–6	580	RID	<10 g/L	8	Ireland
Shivley et al. (18)	2018	1–7	1,623	RID	<10 g/L	12.1	United States
Todd et al. (5)	2018	1–21	2090	ELISA	≤12 g/L	36.2	Ireland
				ZST	≤19 units	64.5	
				TP^2^	≤60 mg/mL	38.8	
				TP	≤5.9 g/dL	40.1	
				BRIX	≤8.4%	30.3	
				Globulin	≤34 g/L	48.6	
Cuttance et al. (53)	2017	1–8	3,819	TP	<52 g/L	33.1	New Zealand
Johnson et al. (84)	2017	1–8	492	RID	<10 g/L	20.7	United Kingdom
				TP	<50 g/L	24.4	
McAloon et al. (10)	2016	1–7	176	TP	52 g/L	32.4	Ireland
				ZST	20 units	42	
				Globulin	20 g/L	8.5	
				GGT	100 IU/L	22.5	
MacFarlane et al. (60)	2015	1–7	392	TP	5.6 g/dL	26	United Kingdom
Windeyer et al. (6)	2014	1–7	2,874	TP	<5.2 g/dL	11	Canada
					<5.7 g/dL	32	
Vogels et al. (57)	2013	1–7	1,018	TP	<5 g/dL	38	Australia
					<4 g/dL	8	
Beam et al. (85)	2009	1–7	1816	RID	<10 g/L	19.2	United States
Trotz-Williams et al. (15)	2008	1–8	933	TP	<5.2 g/dL	8.4	Canada
		1–7	407	TP	<5.2 g/dL	37.1	

^1^RID, Radial immunodiffusion; ELISA, Enzyme linked immunosorbent assay; TP, Total protein; ZST, Zinc sulfate turbidity; GGT, Gamma-glutamyl transferase; ^2^Measured using clinical analyzer; ^3^Age is not available.

A recent large-scale Irish on-farm study found that at least 20% of (suckler) beef and dairy calves had FPT, determined by various testing methods and cut-off values (5). These prevalence rates are comparable to or higher than those reported in North America, Australasia, and other European countries (Table 1). In addition to FPT prevalence, Todd et al. (5) also reported that 20 and 30% of beef and dairy calves, respectively, experienced at least one morbidity event from birth to 6 months of age, with the highest morbidity incidence occurring within the first 30 days of life. Given these concerning rates, there is a clear need to better understand the factors influencing calf immunity and health on Irish farms.

Presently, data on management practices and their associations with passive immune measures and FPT in beef and dairy calves in Ireland are limited. Moreover, no studies have specifically examined risk factors for morbidity in Irish beef and dairy calves. Barry et al. (9) identified herd size, calf age, and breed as risk factors associated with serum IgG concentrations in dairy calves, but they did not assess FPT risk. McAloon et al. (10) reported associations between calving season, time in the calving pen, and colostrum feeding practices with passive immune measures and FPT that were assessed using multiple tests. However, the generalisability of that study is constrained by its small sample size (176 calves) and because it was conducted on a single dairy farm with endemic Johne’s disease (*Mycobacterium avium paratuberculosis*).

International studies have identified dam or calf characteristics (dam parity, calf sex, and breed) and herd-level or calf-level management practices (dam vaccination, calving assistance, time of cow-calf separation, and colostrum interventions) as risk factors for FPT in beef (11–14), and dairy calves (15–18). Several of these risk factors such as dam parity, breed, calving assistance, and colostrum interventions have also been associated with increased calf morbidity (19–21). While these research findings provide valuable insights into FPT and morbidity risk factors in calves, their applicability to pasture-based, seasonal calving production systems in Ireland and elsewhere may be limited. For example, most of the aforementioned studies were conducted in indoor year-round calving production systems or in veal facilities (11, 14–16, 18, 20). In contrast, beef and dairy production in Ireland are predominantly pasture-based systems and typically follow a relatively compact spring-calving pattern, with over 60% of beef calves and 75% of dairy calves born between February and April (22). This condensed calving pattern can create additional challenges for effectively managing calf health and welfare.

The objectives of the present study were to assess both dam and calf characteristics, as well as herd-level and calf-level management practices, associated with: (1) passive immune measures evaluated using various test methods and FPT defined by different cut-off values, and (2) overall morbidity within the first 30 days of life in spring-born Irish beef and dairy calves.

## 2 Materials and methods

### 2.1 Ethical approval

The collection of data used in the present study was approved by the Teagasc Animal Ethics Committee (TAEC18/2013) and Health Products Regulatory Authority (HPRA; Project Authorization No. AE19132-P006), Co. Dublin, Ireland.

### 2.2 Data source

Data for the present study were subsets of a large-scale observational data collected by Todd et al. (5) in spring 2015 and 2016 from herd-level and calf-level studies, respectively. The herd-level study included visits to 92 beef farms (457 calves) and 80 dairy farms (714 calves), with information on farm demographics and herd-level management practices obtained through interviews. The calf-level study included 9 beef farms (406 calves) and 8 dairy farms (957 calves), selected from the herd-level study. The farms were visited during the calving season, and calf-level information on birth and colostrum management were recorded. Information on dam and calf characteristics, passive immune status, farmer-recorded morbidity (e.g., diarrhea, BRD, and other diseases such as navel and joint infections), and mortality were also available. Todd et al. (5) evaluated passive immune status in calves aged 1 to 21 days by analyzing serum samples using multiple methods: ELISA for total IgG (ELISA-IgG), a clinical analyzer for total protein (TP-CA), a digital refractometer for total protein (TP-DR), globulin concentration, zinc sulfate turbidity (ZST), and an optical BRIX refractometer for total solids (TS-BRIX). However, since serum IgG concentrations decline linearly during the first weeks of life (23) and active immunity is not fully functional until after 2 weeks of age (24), assessing passive immune status within the first 14 days may offer a more accurate indication of FPT, and aligns more closely with other published studies (Table 1). Furthermore, among the six passive immune tests evaluated by Todd et al. (5), strong correlations were observed between ELISA-IgG, TP-CA, and TS-BRIX (*r* = 0.77–0.93), supporting their frequent use in the literature (Table 1). Consequently, the present study included only calves whose passive immune status was assessed between 1 and 14 days of age using ELISA-IgG, TP-CA, and TS-BRIX.

### 2.3 Data analysis

#### 2.3.1 Data preparation

**Figure 1:**
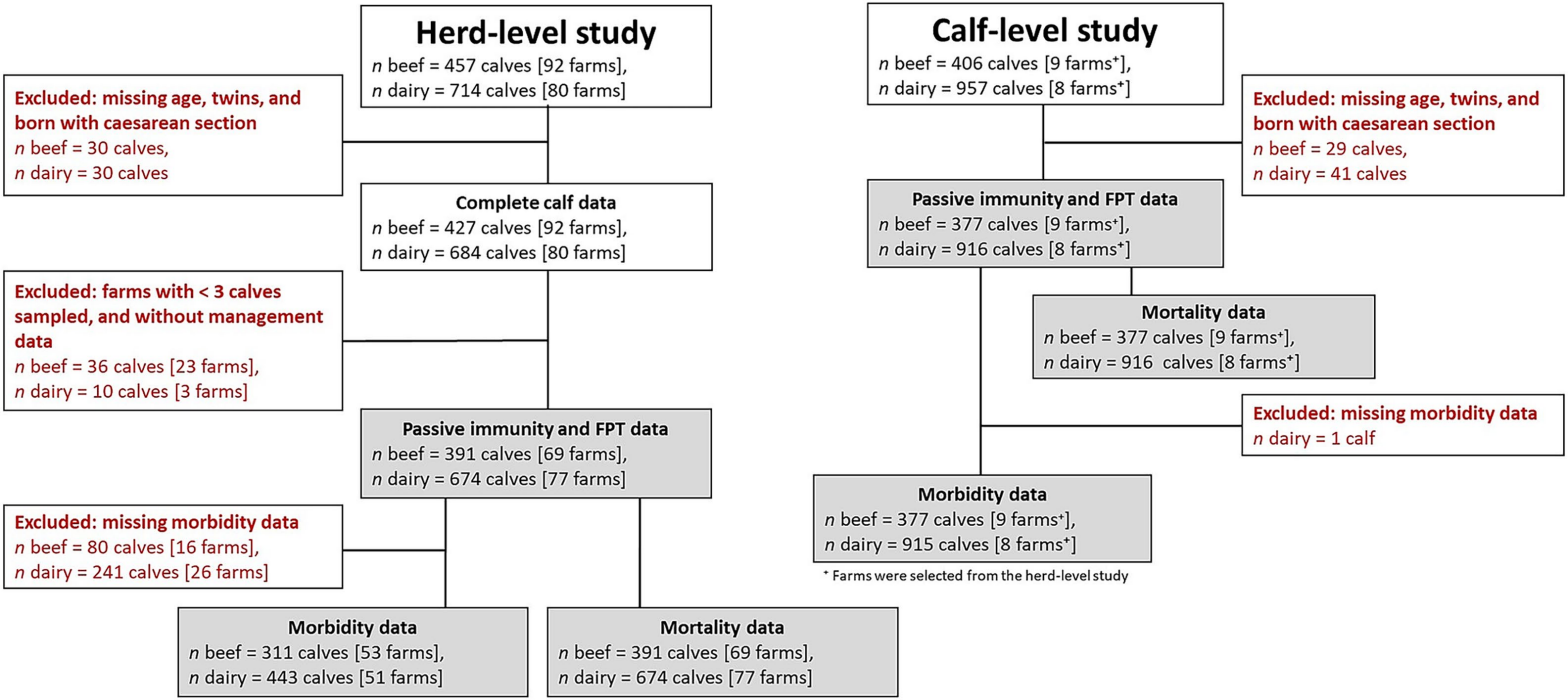
Flow chart of number of calves analyzed from the herd-level and calf-level studies.

The datasets for analysis were prepared separately for the herd-level and calf-level studies due to differences in the information collected in each study. Calves with missing age data, twins, or born via caesarean section were excluded from analysis. For the herd-level study, additional exclusion was applied to remove farms with fewer than three calves sampled or those lacking management practices data (Figure 1). The final dataset from the herd-level study included 69 beef (391 calves) and 77 dairy farms (674 calves), and the calf-level study included 9 beef (377 calves) and 8 dairy farms (916 calves). The final number of calves included per farm in the herd-level study ranged from 3 to 13 beef and 3 to 12 dairy calves, and in the calf-level study the final number ranged from 9 to 90 beef and 73 to 166 dairy calves.

The primary outcomes of interest in the present study were calf passive immune measures obtained from ELISA-IgG, TP-CA, and TS-BRIX, as well as FPT status, and overall calf morbidity during the first 30 days of life. While calf mortality was initially considered as an outcome, it was ultimately excluded from the analysis due to the low number of mortality events observed within the first 30 days both in the herd-level (beef: 0.8%, dairy: 1.7%) and calf-level studies (beef: 0.3%, dairy: 1%). Calf FPT status was defined as a dichotomous outcome (yes/no) based on the passive immunity cut-offs for classification of morbidity or mortality in Irish beef and dairy calves (5), hereafter referred to as “current-cut-offs.” The current-cut-offs of ELISA-IgG were ≤9 and ≤12 mg/mL for beef and dairy calves, respectively, and the TP-CA and TS-BRIX were ≤60 g/L and ≤8.4%, respectively, for both beef and dairy calves. For comparative purposes, FPT was also defined using commonly adopted cut-offs in the published literature (Table 1), hereafter referred to as “literature-cut-offs.” The literature-cut-offs were identical for both beef and dairy calves, with ELISA-IgG <10 mg/mL, TP-CA <52 g/L, and TS-BRIX <8.4%.

Dam and calf characteristic variables from the herd-level and calf-level studies included dam parity, calf sex, breed, birth month, and timing of birth in calving season. Calf breeds were classified according to their predominant genotype. Beef calves were classified as either early-maturing (Aberdeen Angus, Hereford, Shorthorn, and Salers) or late-maturing (Limousin, Charolais, and Simmental). Dairy calves were classified as either pure-dairy (Friesian, Holstein, and Jersey) or dairy-beef, which included the aforementioned beef breeds. Timing of birth in the calving season was classified based on the quartile of calving dates on each farm (first to last date of calf birth during the spring-calving season). Calves born before the first quartile date of the calving season were classified as early, in the second and third quartiles as peak, and in the fourth quartile date as late. The management variables from the herd-level study included farmer employment status (full-time *v*. part-time), pre-calving concentrate feeding, dam diarrhea vaccination pre-calving, cleaning of the dam’s udder after calving, length of dry period, time of removal from the calving pen, time of cow-calf separation (only for dairy calves), practice of navel disinfection, calf pneumonia vaccination, housing location, type of calf pen, group size at housing, age difference between calves in group, and pen cleaning between calf groups. The management variables from the calf-level study included calving area, calving assistance, presence of perinatal problems, calving supervision, and management of first colostrum feeding such as method, time, volume, source, and type of colostrum fed to the calves. The time for first-colostrum feeding was determined by subtracting the time when calves were observed to first suckle from the dam, or received first hand-fed colostrum, from the reported time of birth. Data on the volume of colostrum feeding were only available for calves that were hand-fed colostrum; therefore, a new category called “suckled directly from the dam” was created to account for calves that suckled naturally.

#### 2.3.2 Statistical analysis

Descriptive statistics were generated in SAS 9.4 (SAS Institute Inc., Cary, North Carolina, United States) to summarize passive immunity measures, number of calves with FPT, morbidity, and frequency distribution of variables in herd-level and calf-level studies. Differences in calf-level colostrum management (categorical) between beef and dairy calves, and between male and female dairy calves were evaluated using Chi-square (*χ*^2^) tests. Pearson’s coefficient was used to evaluate the correlations between passive immune measures obtained from ELISA-IgG, TP-CA, and TS-BRIX. Cohen’s Kappa statistic was used to quantify the agreement between FPT defined by current-cut-offs and literature-cut-offs of the three testing methods. The agreement was classified as “fair” (0.20–0.40), “moderate” (0.41–0.60), “substantial” (0.61–0.80), and “almost perfect” (0.81–1.00) (25). The apparent sensitivity (Se), specificity (Sp), positive predictive value (PPV), negative predictive value (NPV), and accuracy of the current-cut-off and literature-cut-off of TP-CA and TS-BRIX to correctly identify calves with FPT were defined using current-cut-off and literature-cut-off of ELISA-IgG as the direct test “standard” references.

Factors associated with passive immune measures (continuous outcome) obtained from ELISA-IgG, TP-CA, and TS-BRIX, and FPT status defined by current-cut-offs and literature-cut-offs of the three testing methods were evaluated in SAS 9.4 using mixed-effect linear and logistic regression models, respectively, with farm included as a random effect. All variables in the herd-level and calf-level studies were evaluated as potential risk factors for passive immune measures and FPT, except several variables from the herd-level study, such as navel disinfection, calf pneumonia vaccination, housing type and location, group size at housing, age difference between calves in group, and pen cleaning between calf groups. Variable categories with fewer than 10 observations were not included in the risk factor analysis to maintain the stability of regression coefficients (26). The model building was similar for mixed-effect linear and logistic regression models. Univariable analysis was initially generated, and variables with *p* < 0.15 were further examined for correlation before being forwarded to multivariable analysis. If strong correlation was observed between a pair of variables (Cramer’s V > 0.7), only the variable with fewest missing data or produce model with the lowest Akaike Information Criterion (AIC) was selected for multivariable analysis (27). Missing data were excluded from multivariable analysis (case-wise removal). Age of calf at sampling was included in all multivariable models, regardless of its significance, to control for declining passive immunity with increased age (23). Final multivariable models were constructed using a manual backward elimination (27), until remaining variables yielded *p* < 0.05 or identified as confounders. Confounding was deemed present if the coefficients of the remaining variables changed by >25% after the elimination of a variable from the model. Two-way interactions between variables with *a priori* biological plausibility were evaluated and retained in the final model if *p* < 0.05. For the final mixed-effect linear models, the assumption of linearity was evaluated by plotting the continuous variable (calf age at sampling) against the outcome variable. Homoscedasticity and normality assumptions were evaluated by assessing the residual plots. A square root transformation was applied to approximately normalize the distribution of passive immune measures obtained from ELISA-IgG in beef (herd-level and calf-level studies) and dairy (herd-level study) calves. Intra-class correlation coefficient (ICC) was calculated using the random effect covariance obtained from the final mixed-effect linear and logistic regression models (27) to account for the variance attributed to the farm as random effect.

Factors associated with overall morbidity in the first 30 days of life were evaluated in a time-to-event survival analysis using Cox proportional hazard models, with farm as a random effect (gamma frailty). The analysis was conducted in R Studio (version 2022.07.2) using the “survival” (28) and “survminer” packages (29). Calves were right censored if exported or lost to follow-up in the first 30 days of life. The survival (non-diseased) probabilities between beef and dairy calves in both herd-level and calf-level studies were examined and visualized using Kaplan–Meier survival curve. All variables from herd-level and calf-level studies, passive immune measures, and FPT defined by current-cut-offs and literature-cut-offs of ELISA-IgG, TP-CA, and TS-BRIX were evaluated as potential risk factors for morbidity. The final model building was similar to that outlined for the mixed-effect linear and logistic regression models. The proportional hazard assumption of each variable in the final Cox proportional hazard model was examined using Schoenfeld residuals. A stratification method was used in the final model if a variable violated the hazard assumption (30).

## 3 Results

### 3.1 Herd-level study

#### 3.1.1 Passive immune measures and FPT

Passive immune measures and prevalence of FPT, defined by current-cut-offs and literature-cut-offs of ELISA-IgG, TP-CA, and TS-BRIX, in beef and dairy calves are summarized in Table 2. Depending on the testing method and cut-off value used, prevalence of FPT ranged from 13.3 to 43.7% and 8.3 to 31.0% in beef and dairy calves, respectively. There were strong positive correlations (*p* < 0.05) between serum passive immune measures obtained from the three testing methods (beef: *r* = 0.72–0.91; dairy: *r* = 0.76–0.92), with “fair” to “almost perfect” Cohen’s Kappa agreement between FPT definitions (beef: 0.33–0.89; dairy: 0.34–0.84) (Supplementary Table 1). There was a substantial variation in apparent sensitivity and specificity of the current-cut-offs and literature-cut-offs of TP-CA and TS-BRIX to correctly identify FPT, defined by current-cut-off and literature-cut-off of ELISA-IgG as the “standard” reference, in beef (Se: 0.36–0.89; Sp: 0.73–0.98) and dairy calves (Se: 0.27–0.91; Sp: 0.81–1.00) (Supplementary Table 2).

**Table 2:** Passive immune measures and prevalence of failure of passive transfer of immunity (FPT), defined by current-cut-offs and literature cut-offs of ELISA-IgG, TP-CA, and TS-BRIX, in beef and dairy calves.

Passive immune tests and cut-offs	Herd-level study		Calf-level study	
	Beef	Dairy	Beef	Dairy
Passive immune measures^1^				
ELISA-IgG, mg/mL	12.4 (5.5)	15.1 (6.2)	13.2 (5.7)	14.1 (5.2)
TP-CA, g/L	61.4 (8.2)	64.0 (8.4)	60.2 (8.4)	62.5 (8.2)
TS-BRIX, %	8.8 (1.0)	9.1 (1.0)	8.8 (0.9)	9.1 (1.0)
Prevalence of FPT (%)				
*Current-cut-offs* ^2^				
ELISA-IgG	27.6	30.0	24.1	32.1
TP-CA	43.7	31.0	49.9	36.9
TS-BRIX	35.3	25.6	34.8	27.6
*Literature-cut-offs* ^3^				
ELISA-IgG	34.5	16.9	27.3	21.3
TP-CA	13.3	8.3	17.5	10.2
TS-BRIX	30.4	20.2	28.4	21.4

^1^Mean (standard deviation); ^2^FPT defined by current-cut-offs (ELISA-IgG ≤9 mg/mL, TP-CA ≤60 g/L, TS-BRIX ≤8.4% for beef; ELISA-IgG ≤12 mg/mL, TP-CA ≤60 g/L, TS-BRIX ≤8.4% for dairy calves); ^3^FPT defined by literature-cut-offs (ELISA-IgG <10 mg/mL, TP-CA <52 g/L, TS-BRIX <8.4% for both beef and dairy calves).

Frequency distribution of variables included in the herd-level study are presented in Table 3. The risk factors associated with passive immune measures and FPT in beef and dairy calves are summarized in Table 4 and Supplementary Tables 3–6. There was no interaction or confounding effect (*p* > 0.05) identified in all final multivariable linear and logistic regression models. Beef calves born on farms that did not immunize the dams against diarrhea pre-calving (*v.* practiced vaccination) had lower passive immune measures based on ELISA-IgG and TS-BRIX, and greater odds for FPT defined by current-cut-offs of all tests and the literature-cut-off of ELISA-IgG. Dairy calves born to primiparous dams (*v.* multiparous) had lower serum TP-CA and TS-BRIX concentrations and were more likely to have FPT defined by current-cut-offs of TP-CA and TS-BRIX only. Male dairy calves (*v.* female) had lower passive immune measures based on TP-CA and greater odds for FPT defined by current-cut-offs and literature-cut-offs of ELISA-IgG and TP-CA. Dairy calves born on farms with a dry period ≤8 weeks (*v.* >8 weeks) had an increased likelihood of FPT defined by all cut-off values except the literature-cut-off of TS-BRIX.

**Table 3:** Frequency distributions of dam, calf, and birth characteristics, and herd-level management practices on 69 beef (391 calves) and 77 dairy farms (674 calves) in the herd-level study.

Variables^1^	Beef				Dairy			
	Farm		Calves		Farm		Calves	
	*n*	%	*n*	%	*n*	%	*n*	%
Dam, calf, and birth characteristics								
Dam parity								
Multiparous			340	87.0			497	74.4
Primiparous			51	13.0			171	25.6
Birth month								
January			92	23.5			142	21.1
February			100	25.6			197	29.2
March			71	18.1			278	41.2
April			125	32.0			51	7.6
May			3	0.8			6	0.9
Timing of birth in the calving season								
Early			114	29.2			177	26.3
Peak			190	48.6			250	37.1
Late			87	22.3			247	36.7
Calf sex								
Female			193	49.4			397	58.9
Male			198	50.6			277	41.1
Breed (beef)								
Early-maturing			51	13.0			N/A	
Late-maturing			340	87.0				
Breed (dairy)								
Pure-dairy			N/A				518	77.5
Dairy-beef							150	22.5
Herd-level management practices								
Farmer employment status								
Full-time	53	76.8	317	81.1	77	100.0	674	100.0
Part-time	16	23.2	74	18.9	–	–	–	–
Dam diarrhea vaccination pre-calving^2^								
No	38	55.1	191	48.9	23	29.9	204	30.3
Yes	31	44.9	200	51.2	54	70.1	470	69.7
Concentrate feeding pre-calving								
No	58	84.1	330	84.4	47	61.0	412	61.1
Yes	11	15.9	61	15.6	30	39.0	262	38.9
Length of dry period								
>8 weeks	62	91.2	351	92.6	52	67.5	463	68.7
≤8 weeks	6	8.8	28	7.4	25	32.5	211	31.3
Heifers calving early in the calving season								
Always	11	16.2	59	15.2	11	14.3	103	15.3
Sometimes	37	54.4	228	58.8	37	48.1	333	49.4
Never	20	29.4	101	26.0	29	37.7	238	35.3
Time of removal from calving pen								
Within 24 h	15	21.7	90	23.0	62	83.8	549	85.0
>24 h	53	76.8	297	76.0	12	16.2	97	15.0
Calve outdoors	1	1.5	4	1.0	–	–	–	–
Clean dam’s udder after calving								
No	38	55.9	218	57.5	36	48.0	300	45.6
Yes	30	44.1	161	42.5	39	52.0	358	54.4
Time of cow-calf separation after birth								
Within 2 h	N/A				26	34.7	225	34.2
>2 h					49	65.3	433	65.8
Mineral and/or vitamin bolus in 24 h after birth								
Always	2	2.9	11	2.8	2	2.63	15	2.26
Never	64	92.8	361	92.3	74	97.37	648	97.74
Sometimes	3	4.4	19	4.9	–	–	–	–
Navel disinfection after birth								
No	3	4.4	20	5.1	7	9.2	53	8.0
Yes	66	95.7	371	94.9	69	90.8	610	92.0
Calf pneumonia vaccination								
No	46	66.7	263	67.3	62	80.5	546	81.0
Yes	23	33.3	128	32.7	15	19.5	128	19.0
Type of calf pen								
Group hutches up to weaning	N/A				1	1.4	8	1.2
Group pens up to weaning					39	52.7	339	52.2
Individual pen and then group pens					29	39.2	255	39.3
Individual hutch and then group pens					1	1.4	8	1.2
Other^3^					4	5.4	39	6.0
Housing location for cow-calf units (beef) or calves (dairy)								
Same house as other cattle group	28	41.2	153	39.4	16	21.6	125	19.3
Separate house for cow-calf units or calves only	35	51.5	207	53.4	43	58.1	405	62.4
Other^4^	5	7.3	28	7.2	15	20.3	119	18.3
Group size of beef cow-calf units at housing								
15 or more units/group	21	35.0	104	30.7	N/A			
12 to 14 units/group	7	11.7	40	11.8				
<12 units/group	31	51.7	191	56.3				
Individually	1	1.7	4	1.2				
Group size of dairy calves at housing								
15 or more calves/group	N/A				17	23.3	164	25.6
12 to 14 calves/group					11	15.1	94	14.7
<12 calves/group					45	61.6	383	59.8
Age difference between calves in group								
3 weeks or more	38	66.7	185	58.4	19	25.7	176	27.1
<3 weeks	19	33.3	132	41.6	55	74.3	473	72.9
Pen cleaning between groups of cow-calf units (beef) or calves (dairy)								
Yes	2	3.2	14	4.0	13	18.6	111	18.3
No	60	96.8	340	96.1	57	81.4	497	81.7

N/A – Not applicable; ^1^Sums for each variable varied due to missing data; ^2^Vaccination against coronavirus, rotavirus, *Escherichia coli (E. coli)*, and salmonella; ^3^Other: group pens then group hutches or vice versa; ^4^Other: house in multiple locations or outdoor.

**Table 4:** Summary of risk factors associated with passive immune measures and failure of passive transfer of immunity (FPT), defined by current-cut-offs and literature-cut-offs of ELISA-IgG, TP-CA, and TS-BRIX, in beef and dairy calves in the herd-level and calf-level studies.

Variables	Beef				Dairy			
	ELISA-IgG	TP-CA	TS-BRIX	Odds ratio^1^	ELISA-IgG	TP-CA	TS-BRIX	Odds ratio
Herd-level study^2^								
Dam parity (primiparous *v.* multiparous)	NS	NS	NS	–	NS	P^↓^, CC	P^↓^, CC	1.66 to 1.95^4^
Calf sex (male *v.* female)	NS	NS	NS	–	CC, LC	P^↓^, CC, LC	NS	1.48 to 2.39
Dam diarrhea vaccination pre-calving (no *v.* yes)	P^↓^, CC, LC	CC	P^↓^, CC	1.70 to1.97	NS	NS	NS	–
Length of dry period (≤8 *v.* >8 weeks)	NS	NS	NS	–	CC, LC	CC, LC	CC	1.74 to 2.10
Calf-level study^3^								
Timing of birth in the calving season (peak *v.* early)	P^↓^, CC, LC	NS	NS	2.52 to 3.84	NS	NS	NS	–
Dam parity (primiparous *v.* multiparous)	NS	CC	NS	3.10	P^↓^, CC, LC	P^↓^, CC	P^↓^	1.50 to 1.59
Breed (sucker beef: late- *v.* early-maturing; dairy: dairy-beef *v.* pure-dairy)	CC	NS	NS	3.17	NS	CC	CC	1.47 to 1.55
Calving area (group *v.* individual calving pen)	NS	CC	NS	0.28	NS	LC	NS	6.71
Calving supervision (not supervised *v.* present for calving)	NS	NS	NS	–	CC, LC	CC, LC	CC, LC	0.58 to 0.71
Perinatal problems (yes *v.* no)	P^↓^, CC, LC	P^↓^, CC, LC	P^↓^, CC, LC	3.63 to 4.53	NS	P^↓^	NS	–
Time of first colostrum feeding after birth (>2 h *v.* within 2 h post-birth)	P^↓^, CC, LC	P^↓^, CC, LC	P^↓^, LC	2.34 to 10.12	NS	NS	NS	–
Method of colostrum feeding (suckled dam with assistance *v.* without assistance)	NS	LC	NS	2.99	NS	NS	NS	–
Type of colostrum feeding (stored *v.* fresh colostrum)	NS	NS	NS	–	NS	LC	LC	2.76 to 5.02

P^↓^ – Lower passive immune measure; CC – FPT defined by current-cut-offs; LC – FPT defined by literature-cut-offs; NS – Not significant (*p* > 0.05); ^1^Odds ratio for FPT; ^2^Variables dam diarrhea vaccination pre-calving and dry period length were recorded at herd-level; ^3^All variables were recorded at calf-level; ^4^Example interpretation: Dairy calves born to primiparous dams had lower passive immune measures based on TP-CA and TS-BRIX, and 1.66 to 1.95 times greater odds for FPT defined by current-cut-offs of TP-CA and TS-BRIX, than those born to multiparous.

#### 3.1.2 Morbidity

**Figure 2:**
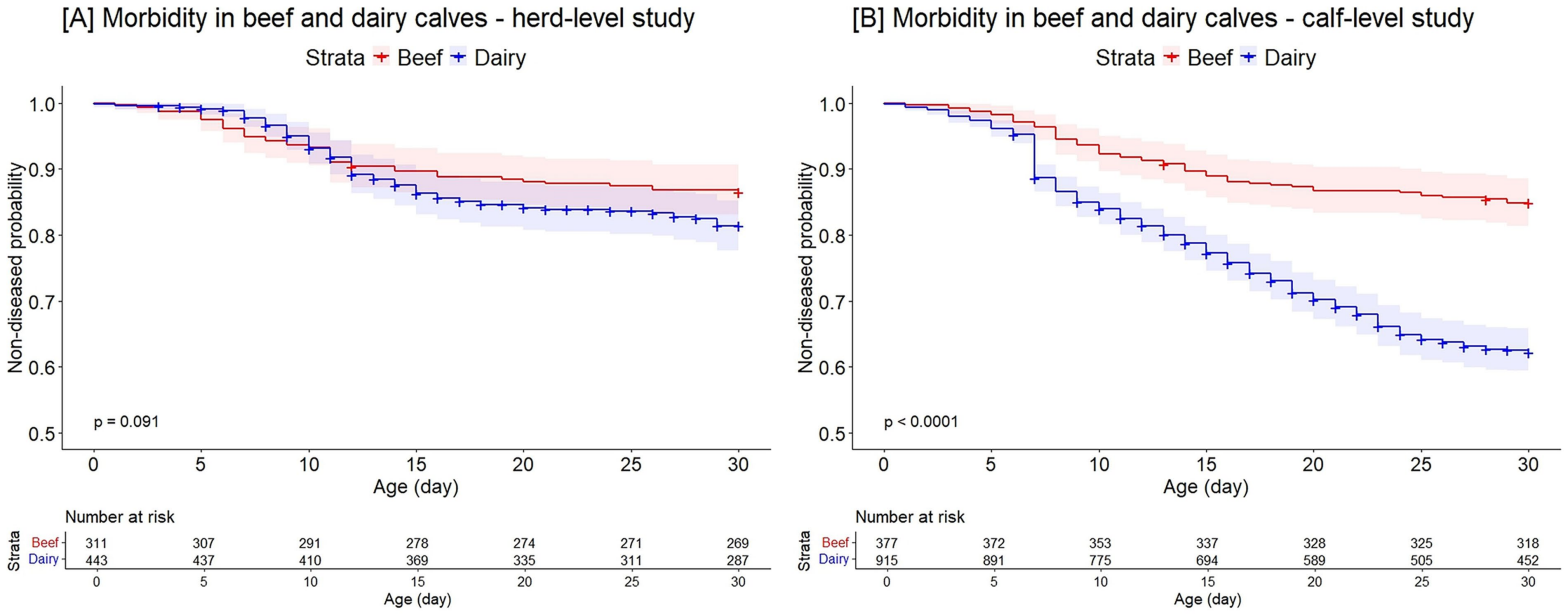
Kaplan–Meier curve of the probability of not having a morbidity event during the first 30 days of life between beef and dairy calves in the herd-level **(A)** and the calf-level study **(B)**.

In the first 30 days of life, 13% of beef calves experienced at least one, and 9.5% of these calves had more than one, morbidity event. The proportion of beef calves with diarrhea, BRD, and other diseases was 7.4, 1, and 5.5%, respectively. In the first 30 days of life, 17.6% of dairy calves had at least one, and 5.1% of these calves had more than one, morbidity event. The proportion of diarrhea, BRD, and other disease events in dairy calves was 15.8, 1.1, and 0.7%, respectively. The probability of not having a morbidity event by day 30 of life was 87 and 81% for beef and dairy calves, respectively (*p* = 0.09; Figure 2).

Risk factors associated with morbidity in the first 30 days of life in beef and dairy calves are summarized in Table 5. In beef calves, none of the passive immune measures were associated with morbidity (*p* > 0.05). However, the hazard for morbidity was greater in beef calves with FPT, defined by current-cut-off and literature-cut-off of TS-BRIX, or in calves reared on farms where cow-calf units were housed with other cattle groups as opposed to being housed separately. Beef calves born to primiparous dams had lower morbidity hazard than those born to multiparous dams. The morbidity hazard was greater in dairy calves reared on farms where cow-calf separation occurred more than 2 h post-birth (*v.* within 2 h) and on farms that did not clean pens between calf groups (*v.* clean pens). Passive immune measures and FPT status, defined by current-cut-offs and literature-cut-offs of all passive immune tests, were not associated (*p* > 0.05) with morbidity in dairy calves.

**Table 5:** Final Cox regression models of risk factors associated with morbidity in the first 30 days of life in beef and dairy calves in the herd-level study.

Variables	Category	*β*	Hazard ratio	CI 95%	*p*-value
Beef^1^					
FPT defined by current-cut-off of TS-BRIX^2^	No (>8.4%)	Ref.	Ref.	Ref.	Ref.
	Yes (≤8.4%)	1.12	3.08^6^	1.43–6.61	0.004
Dam parity	Multiparous	Ref.	Ref.	Ref.	Ref.
	Primiparous	−1.50	0.22	0.05–0.96	0.044
Housing location for cow-calf units^3^	Separate house for cow-calf unit only	Ref.	Ref.	Ref.	Ref.
	Same house as other cattle group	1.77	5.87	1.88–18.31	0.002
Random effect (farm)					0.003
Dairy^4,5^					
Time of cow-calf separation after birth	Within 2 h	Ref.	Ref.	Ref.	Ref.
	>2 h	1.94	6.95	1.34–35.93	0.021
Pen cleaning between calf groups	Yes	Ref.	Ref.	Ref.	Ref.
	No	2.47	11.84	1.59–88.34	0.016
Random effect (farm)					<0.001

^1^*n =* 296 calves; ^2^FPT defined by literature-cut-off of TS-BRIX (<8.4%) was also associated with increased morbidity hazard (Hazard ratio 2.76; CI 95% 1.30–5.84); ^3^Variable was recorded at herd-level; ^4^*n =* 395 calves; ^5^All variables were recorded at herd-level; ^6^Example interpretation: Beef calves with FPT, defined by current-cut-off of TS-BRIX, had a 3.08 times greater hazard for morbidity in the first 30 days of life than those without FPT.

### 3.2 Calf-level study

#### 3.2.1 Passive immune measures and FPT

The concentrations of passive immune measures and prevalence of FPT, defined by current-cut-offs and literature-cut-offs of ELISA-IgG, TP-CA, and TS-BRIX, in beef and dairy calves are summarized in Table 2. Prevalence of FPT varied by testing methods and cut-off values, ranging from 17.5 to 49.9% in beef and 10.2 to 36.9% in dairy calves. Strong positive correlations (*p* < 0.05) were observed between passive immune measures obtained from ELISA-IgG, TP-CA, and TS-BRIX, with correlation coefficients ranging from 0.80 to 0.95 in both beef and dairy calves. The Cohen’s Kappa agreement between FPT definitions was “fair” to “almost perfect” (beef: 0.35–0.92; dairy: 0.32–0.83) (Supplementary Table 1). When using the current-cut-off and literature-cut-off of ELISA-IgG as the “standard” reference, the apparent sensitivity and specificity of the current-cut-offs and literature-cut-offs of TP-CA and TS-BRIX for identifying FPT varied widely in beef (Se: 0.60–0.96; Sp: 0.65–0.99) and dairy calves (Se: 0.32–0.93; Sp: 0.78–1.00) (Supplementary Table 2).

Descriptive data for variables in the calf-level study are summarized in Table 6. Significant differences were observed between beef and dairy calves in the method, source, type, and volume of first colostrum feeding (*p* < 0.0001), with no difference in time of first colostrum feeding (*p* = 0.32; Supplementary Table 7). There was no difference in colostrum management practices between male and female dairy calves (*p* > 0.05; Supplementary Table 7). The risk factors associated with passive immune measures and FPT in beef and dairy calves are summarized in Table 4 and Supplementary Tables 8–11. There was no interaction or confounding effect identified in all final multivariable linear and logistic regression models (*p* > 0.05). Beef calves born during the peak calving season (*v.* early) had lower passive immune measures based on ELISA-IgG and greater odds for FPT defined by the current-cut-off and literature-cut-off of ELISA-IgG only. Beef calves born to primiparous dams (*v.* multiparous) or late-maturing breeds (*v.* early-maturing) were more likely to have FPT defined by current-cut-offs of TP-CA and ELISA-IgG, respectively. The presence of perinatal problems (*v.* none) in beef calves was associated with lower passive immune measures based on all tests, and a greater likelihood of having FPT defined by all cut-off values. Beef calves that consumed colostrum more than 2 h post-birth (*v.* within 2 h) had lower passive immune measures based on all tests and greater odds for FPT defined by all cut-off values except the current-cut-off of TS-BRIX. The odds of FPT, defined by literature-cut-off of TP-CA, were greater in beef calves that suckled the dam with assistance (*v.* without assistance). Beef calves born in group calving pens (*v.* individual) were less likely to have FPT defined by current-cut-off of TP-CA, whereas the odds for FPT defined by literature-cut-off of TP-CA were greater in dairy calves born in group calving pens. In dairy calves, perinatal problems were only associated with lower serum TP-CA concentrations. Dairy calves born to primiparous (*v.* multiparous) had lower passive immune measures based on all tests and a greater likelihood of FPT defined by the current-cut-offs of ELISA-IgG and TP-CA, and the literature-cut-off of ELISA-IgG. The odds of FPT, defined by current-cut-offs of TP-CA and TS-BRIX, were greater in dairy-beef than pure-dairy calves. In dairy calves, the likelihood for FPT defined by all cut-off values was lower when calving was not supervised (*v.* present at calving). Feeding stored colostrum (*v.* fresh) also increased the likelihood of FPT, defined by the literature-cut-offs of TP-CA and TS-BRIX, in dairy calves.

**Table 6:** Frequency distributions of dam, calf, and birth characteristics, and calf-level colostrum management practices in 377 beef and 916 dairy calves in the calf-level study.

Variables^1^	Beef		Dairy	
	*n*	%	*n*	%
Dam, calf, and birth characteristics				
Dam parity				
Multiparous	312	82.8	668	73.1
Primiparous	65	17.2	246	26.9
Birth month				
January	38	10.1	165	18.0
February	108	28.6	477	52.1
March	171	45.4	188	20.5
April	58	15.4	83	9.1
May	2	0.5	3	0.3
Timing of birth in the calving season				
Early	128	34.0	273	29.8
Peak	169	44.8	442	48.3
Late	80	21.2	201	21.9
Calf sex				
Female	204	54.1	479	52.4
Male	173	45.9	435	47.6
Breed (beef)				
Early-maturing	50	13.3	N/A	
Late-maturing	327	86.7		
Breed (dairy)				
Pure-dairy	N/A		740	81.0
Dairy-beef			174	19.0
Calving area				
Individual calving pen	151	40.1	204	22.4
Group calving pen	192	50.9	568	62.3
Other^2^	34	9.0	140	15.4
Calving supervision				
Someone presents for calving	160	42.4	491	53.9
Calving not supervised	217	57.6	420	46.1
Calving assistance				
Calved on own	318	84.4	754	82.3
Assisted calving	59	15.7	162	17.7
Perinatal problems				
No	351	93.1	893	97.9
Yes^3^	26	6.9	19	2.1
Calf-level colostrum management				
Time of first colostrum feeding post-birth				
>2 h	41	19.5	154	22.8
Within 2 h	169	80.5	521	77.2
Method of colostrum feeding				
Suckled dam without assistance	276	73.2	168	18.5
Suckled dam with assistance	76	20.2	2	0.2
Hand-fed	25	6.6	739	81.3
Method of colostrum feeding if hand-fed				
Bucket	–	–	76	10.3
Bucket with teat	–	–	23	3.1
Nipple bottle	6	24.0	412	55.9
Stomach tube	19	76.0	226	30.7
Volume of first colostrum feeding				
3 L or More	–	–	459	53.6
<3 L	25	6.6	227	26.5
Suckled directly from dam	352	93.4	170	19.9
Volume of first colostrum if hand-fed	1.5 to 2 L (mean 2, SD 0.1, median 2 L, *n =* 25)		0.5 to 4.5 L (mean 2.8, SD 0.4, median 3 L, *n* = 686)	
Total volume of colostrum in the first 24 h				
6 L or more	–	–	265	31.5
<6 L	15	4.1	406	48.3
Suckled directly from dam	352	95.9	170	20.2
Total volume of colostrum in the first 24 h if hand-fed	2 to 4 L (mean 2.1, SD 0.5, median 2 L, *n =* 15)		2 to 12 L (mean 5.4, SD 1.5, median 5 L, *n* = 671)	
Source of colostrum				
From dam only^4^	359	95.2	575	63.3
From donor cow/cows	–	–	334	36.7
Artificial colostrum	4	1.1	–	–
Obtained from another herd	14	3.7	–	–
Type of colostrum				
Freshly harvested^5^	12	3.2	704	77.5
Stored colostrum (refrigerated or frozen)	13	3.5	35	3.9
Suckled directly from dam	352	93.4	170	18.7

N/A – Not applicable; ^1^Sums for each variable varied due to missing data; ^2^Other: outdoors, dry cow pen, cubicles, handling crush; ^3^Perinatal problems: weak, no-suckle responses, dullness, being reluctant to stand, standing difficulty, or miss-mothering; ^4^Harvested colostrum or suckled directly from dam; ^5^Including artificial colostrum.

#### 3.2.2 Morbidity

In the first 30 days of life, 15.1% of beef calves had at least one, and 3.5% of these calves had more than one, morbidity event. The proportion of beef calves with diarrhea, BRD, and other diseases was 10.1, 1.6, and 4.2%, respectively. Among dairy calves, 36% experienced at least one, and 3.3% of these calves had more than one, morbidity event in the first 30 days of life. The proportion of diarrhea, BRD, and other diseases was 30.4, 3.6, and 3.2%, respectively. The probability of not having a morbidity event by day 30 of life was higher in beef than dairy calves (85% *v.* 62%, *p* < 0.0001; Figure 2).

In the final multivariable Cox proportional hazard model, no variable was associated with morbidity in beef calves (*p* > 0.05). Passive immune measures and FPT status, defined by current-cut-offs and literature-cut-offs of all passive immune tests, were not associated with morbidity in the first 30 days of life in beef and dairy calves (*p* > 0.05), although beef calves with FPT defined by the current-cut-off of TS-BRIX had a tendency for greater morbidity hazard (Hazard ratio 1.65, CI 95% 0.94–2.90, *p* = 0.083). In dairy calves, the final model (Table 7) was stratified using the timing of birth in the calving season variable due to violation of the proportional hazard assumption. The hazard of morbidity was lower in dairy calves born to primiparous than multiparous dams. Dairy calves of dairy-beef breed (*v.* pure-dairy), born with assisted calving (*v.* calved on own) and received hand-fed colostrum (*v.* suckled dam on its own) had greater morbidity hazard in the first 30 days of life.

**Table 7:** Final Cox proportional hazard model^1^ of risk factors associated with morbidity during the first 30 days of life in dairy calves in the calf-level study.

Variables^2,3^	Category	*β*	Hazard ratio	CI 95%	*p*-value
Dam parity	Multiparous	Ref.	Ref.	Ref.	Ref.
	Primiparous	−0.38	0.68	0.52–0.90	0.007
Breed	Pure-dairy	Ref.	Ref.	Ref.	Ref.
	Dairy-beef	0.45	1.56^4^	1.13–2.16	0.007
Calving assistance	Calved on own	Ref.	Ref.	Ref.	Ref.
	Assisted calving	0.41	1.50	1.10–2.04	0.010
Method of colostrum feeding	Sucked dam on its own	Ref.	Ref.	Ref.	Ref.
	Hand-fed	0.49	1.64	1.15–2.33	0.006
Random effect (farm)					<0.001

^1^Stratified by timing of birth in the calving season variable; ^2^*n* = 906 calves; ^3^All variables were recorded at calf-level; ^4^Example interpretation: Dairy-beef calves had a 1.56 times greater hazard for morbidity in the first 30 days of life than pure-dairy calves.

## 4 Discussion

### 4.1 Effect of passive immune test and cut-off values on FPT risk factors

The use of multiple testing methods and cut-off values in the present study enabled a comprehensive evaluation of passive immune measures and FPT, and their associated risk factors. The findings indicated that risk factors associated with passive immune measures and FPT were dependent on the testing methods and cut-offs applied, consistent with previous studies in Ireland (10) and Canada (14). Additionally, different risk factors were identified in beef and dairy calves. These results further support the concerns raised by McGee and Earley (31) of the limitations and potential misinterpretations associated with comparing FPT outcomes across different testing methods and cut-off criteria. The variability in identified risk factors associated with passive immune measures and FPT may be attributed to differences in performance characteristics of testing methods and cut-offs used to assess passive immunity and define FPT. Despite the strong positive correlations observed among passive immune measures obtained from ELISA-IgG, TP-CA, and TS-BRIX, the Cohen’s Kappa agreement between FPT status, defined by current-cut-offs and literature-cut-offs of the three testing methods, varied from “fair” to “almost perfect” for beef and dairy calves in both the herd-level (0.33–0.89) and calf-level studies (0.32–0.92). These findings align with Hogan et al. (32) who reported “moderate” to “substantial” agreement (0.59–0.72) when comparing different FPT cut-offs of serum IgG (<10 and <8 mg/mL; based on ELISA) and TP (<52 and <45 g/L; clinical analyzer) in dairy calves. In beef calves, “substantial” to “almost perfect” agreement (0.62–0.89) was evident when comparing various FPT cut-offs of serum TP (ranged from 51 to 57 g/L; digital and optical refractometer) and BRIX (ranged from 7.9 to 8.7%; digital refractometer) (33). From a practical perspective, this variability suggests that a calf could be classified as experiencing FPT under one cut-off yet considered to have adequate passive immunity under another. Such inconsistencies may lead to misclassification, potentially introducing bias in the identification of risk factors and increasing the likelihood of incorrectly attributing causality (34).

The observed ranges of apparent sensitivity and specificity in the present herd-level (0.27–0.91 and 0.73–1.00, respectively) and calf-level studies (0.32–0.96 and 0.65–1.00, respectively) for the current-cut-offs and literature-cut-offs of TP-CA and TS-BRIX to detect FPT (respective ELISA-IgG as the “standard” reference) in beef and dairy calves, were greater than those reported in previous international studies, likely reflecting the wide range of cut-offs evaluated, particularly for TP-CA (ranged from 52 to 60 g/L). For example, Pisello et al. (35) evaluating cut-offs for TP (ranged from 51 to 52 g/L; digital and optical refractometer) and BRIX (8.3%; digital and optical refractometer) in beef calves reported sensitivity and specificity ranges of 0.63–0.77 and 0.90–0.96, respectively (serum IgG <16 mg/mL as reference; RID). In contrast, Denholm et al. (36) evaluating cut-offs for TP (ranged from 50 to 52 g/L; optical refractometer) and BRIX (ranged from 8.2 to 8.4%; optical refractometer) in dairy calves reported sensitivity and specificity ranges of 0.52–0.77 and 0.66–0.86, respectively (serum IgG <10 g/L as reference; RID). Collectively, the findings from the present and previous studies clearly demonstrate that cut-off values significantly affect the performance of passive immune tests in detecting FPT.

Although dichotomous FPT cut-offs have been widely adopted worldwide (5–7), limitations such as potential data loss or distortion (21) and misclassification of calf health outcome, particularly in borderline cases (37), are of concern. To overcome this, Lombard et al. (37) recommended classifying dairy calf serum IgG concentration (and its equivalent TP and total solids), into categories of “poor,” “fair,” “good,” and “excellent” based on the association with morbidity and mortality risks. Based on RID serum IgG, they found 12, 27, 26 and 35% of calves in the “poor,” “fair,” “good,” and “excellent” categories, respectively. Using the Lombard et al. (37) classification in the present herd-level and calf-level studies, 17–21% of dairy calves were classified as having “poor” ELISA serum IgG concentration, with 54–56% classified as “fair,” 22–27% as “good,” and only 1–3% as “excellent.” This discrepancy across studies may be due to differences in management and environmental conditions or in how the serum IgG concentrations were measured. Although serum IgG quantification is widely regarded as the “standard” reference (1, 4), ‘absolute’ concentrations obtained can vary depending on the method used; for example, the concentration of IgG measured using RID can be greater by up to two-fold than that measured by ELISA (38, 39). As highlighted by Michelsen et al. (40), RID-based cut-offs may not be appropriate for ELISA results. Therefore, it would be necessary to establish categories specific to ELISA-derived IgG values.

### 4.2 Risk factors associated with passive immune measures and FPT

In the present herd-level study, the greater FPT odds in dairy calves born on farms with a dry period ≤8 weeks is likely due to reduced colostrum yield and quality in cows with shorter or no dry periods (41–45), particularly since colostrogenesis and the accumulation of immunoglobulins in mammary secretions typically occur during the final weeks of gestation (46). The positive effect of pre-calving dam diarrhea vaccination on passive immune measures of beef calves concurs with Pisello et al. (13), showing higher serum total IgG concentration in beef calves born to dams vaccinated against *E. coli*, rotavirus, and coronavirus. A recent Canadian study (14) found no effect of dam vaccinations on serum total IgG concentration; however, *E. coli* and rotavirus-specific IgG was significantly greater in calves born to multiparous dams vaccinated against *E.coli* and rotavirus. Although the benefits of pre-calving diarrhea vaccination in dams are well established, over 50% of the beef farms included in the present herd-level study did not administer pre-partum vaccinations. This finding underscores the need to promote and encourage wider adoption of this preventive practice on Irish beef farms.

In the present calf-level study, the lower passive immune measure and increased odds of FPT observed in beef calves born during peak calving season may be attributed to heightened labor demands. Fallon et al. (47) found that labor demands for animal husbandry tasks, including the calving and monitoring of cows, on Irish beef farms were highest during the peak calving season. In contrast, the lack of association in the present calf-level study between timing of birth in the calving season and passive immunity in dairy calves, concurs with Barry et al. (9) who reported comparable serum IgG concentrations in calves born in weeks 1–6 and weeks 6–12 of the calving season. The conflicting findings between beef and dairy calves is difficult to interpret, particularly given that labor demands for cow and calf care are also increased on Irish dairy farms during the peak calving season (48). One possible explanation is the typically larger size of dairy operations compared to beef farms (49), which may enable dairy farms to implement better management strategies such as hiring additional full-time or part-time staff in preparation for the peak calving period (48).

The lower FPT odds observed in beef calves born in group pens compared to individual pens in the present calf-level study may be due to the presence of multiple animals, which could stimulate calf suckling behavior through observing and mimicking the behaviors of other calves (50). In dairy calves, McAloon et al. (10) found no difference in passive immunity between calves born in individual pens and those born in non-designated calving areas. However, the present calf-level study identified greater odds of FPT in dairy calves born in group pens. Similarly, Michanek and Ventorp (51) found that dairy calves born in group pens had lower IgG concentrations compared to those born in individual pens. They attributed this finding to cross-suckling behavior commonly observed in group settings, which may reduce colostrum intake if newborn calves suckle from already-suckled cows or if older calves consume colostrum intended for newborns. However, this explanation may not be fully applicable to the current study, since only 18.5% of dairy calves were left to suckle from their dams. Further research is needed to better understand the relationship between the type of calving area and calf passive immunity.

Consistent with findings from previous studies on both beef (12, 14, 52) and dairy calves (53, 54), the present study observed lower passive immune measures and greater odds of FPT in calves born to primiparous dams compared to those from multiparous dams. This outcome is likely attributable to differences in colostrum yield and/or quality, which generally improves with increasing parity (31, 41, 43, 45, 52, 54). Additionally, Brereton et al. (52) reported that under “natural suckling” conditions, primiparous beef dams expressed maternal inexperience, such as less frequent calf licking, and their calves were less vigorous than multiparous beef dams. The latter explanation may partially support the finding from the present calf-level study, as over 90% of beef calves suckled “naturally” from the dam.

In their review, Lorenz et al. (55) highlighted that good calving supervision, such as being present during stage two of calving, is important for preventing FPT or perinatal death in calves. Counterintuitively, in the present calf-level study, supervision at calving appeared to negatively impact passive immunity in dairy calves. While supervisions could be associated with providing assistance in difficult calving, this explanation is unlikely in the present context, as over 50% of dairy calves were born under supervision. Another possible explanation may lie in the potential for unnecessary interventions or environmental disturbances during supervised calving which could prolong the natural calving process and increase the risk of dystocia (56) or metabolic acidosis (1), thereby adversely affect the timely ingestion or absorption of colostral IgG.

The greater FPT odds in late-maturing compared to early-maturing breed beef calves in the present calf-level study contrasts with Waldner and Rosengren (11) and Bragg et al. (12) who reported comparable serum IgG concentration and FPT risk in beef calves from these breed types. The greater odds of FPT observed for dairy-beef compared to pure-dairy calves concurs with Vogels et al. (57), who reported greater risk of agammaglobulinemia in dairy-beef relative to Holstein-Friesian calves. Possible explanations for these findings include breed-related differences in colostral IgG concentrations (41), or the increased risk of calving difficulty or assistance in late-maturing beef breeds (58) and beef-sired dairy cows (*v.* dairy-sired) (59), which has been reported to negatively affect passive immunity in beef (11, 12) and dairy calves (16, 60).

In agreement with previous studies (9, 40, 60), the present calf-level study found no significant differences in passive immune measures and FPT odds between male and female dairy calves. This likely reflects similar colostrum management practices applied to both sexes. However, these findings contrast with those of the present herd-level study and other research (16, 57) which reported lower serum TP concentration and greater risk of agammaglobulinemia in male dairy calves. In their study, Renaud et al. (16) reported variations in colostrum management practices, with a greater proportion of male calves receiving pooled colostrum and being fed colostrum via combination of nipple bottle followed by stomach tube, and they were more likely to receive lower colostrum volume than their female counterparts. Although the variation in passive immune measures and FPT odds may indicate differences in colostrum management between male and female dairy calves in the present herd-level study, this could not be confirmed due to the lack of detailed colostrum management data.

In the present calf-level study, the reduction of passive immune measures and increased odds of FPT in beef calves with delayed colostrum feeding (>2 h post-birth) confirms the time-dependent decline in colostral IgG absorption (1), and further reinforces the importance of ensuring calves consume colostrum immediately after birth. Consistent with the present calf-level study, previous research reported greater odds of FPT in beef calves receiving assistance with colostrum feeding, such as being guided to suckle from the dam or fed colostrum by nipple bottle or tube, compared to those that suckled naturally (12, 14). Unlike dairy calves, which are mostly hand-fed colostrum, beef calves only receive feeding assistance if they have perinatal problems such as a weak suckle reflex, poor vigor, or unable to suckle the dam naturally (61). Optimal colostrum consumption in beef calves depends on their ability to stand, walk, locate the teat, and suckle as soon as possible after birth; therefore, any disruption in these steps can negatively impact passive immunity (31). This is confirmed by findings from the present calf-level study, showing that beef calves with perinatal problems (e.g., weak, reluctant to stand, or no-suckle response) had reduced passive immune measures and increased odds for FPT. Likewise, a Canadian study also found reduction in serum IgG concentration in beef calves with incomplete tongue withdrawal or weak suckling reflex on calf vigor assessment (62). In the present calf-level study, all beef calves with perinatal problems were either hand-fed or received assistance at suckling, with the majority being fed colostrum within 2 h post-birth. While this reflects the farmer’s efforts to support calves with perinatal problems, the assistance provided was insufficient to ensure adequate passive immunity. This further highlights the need for additional measures, such as early assessment of the calf’s suckling reflex (52), to improve the success of colostrum interventions in beef calves with perinatal problems.

The increased odds for FPT in dairy calves that consumed stored (refrigerated or frozen) colostrum in the present calf-level study may be attributed to a decline in colostrum quality and possibly bacterial contamination during storage, freezing, or thawing (42), which can impair IgG absorption in newborn calves (2).

### 4.3 Risk factors associated with morbidity

In the present calf-level study, the lower probability of beef calves experiencing a morbidity event during the first 30 days of life compared to dairy calves is likely due to differences in environmental and management conditions, in that dairy herds are suggested to have higher confinement density than beef herds, which may increase the risk of pathogen exposure (37). Under field conditions, numerous studies have reported that passive immune measures and FPT status are associated with morbidity in both beef (7, 11, 13, 21) and dairy calves (6, 8, 63–65). However, contrary to expectations, the present study found no association between passive immune measures and morbidity in either beef or dairy calves, despite evidence suggesting an increased morbidity hazard in beef calves with FPT defined by current-cut-off and literature-cut-off for TS-BRIX. This finding further highlights the complexity and multifactorial nature of morbidity occurrence, whereby FPT is only one of the contributing factors (5, 6). The discrepancy between results from the present study and previous research may be due to differences in testing methods and cut-off values for assessing passive immune measures and FPT (as discussed earlier), and variations in age-related risk groups whereby morbidity outcomes were recorded and analyzed. For example, previous studies evaluated the impact of passive immune measures and/or FPT on morbidity in beef calves from birth to 3 months old (11) or up to 6 months old (7, 13), and in dairy calves from birth to 2 months (64), birth to 3 months old (6, 65), or during the fattening period in veal facilities (63). In contrast, the present study limited morbidity assessment to the first 30 days of life, the period when morbidity incidence was highest (5), and to reduce potential confounding factors influencing the relationship between FPT and morbidity. Overall, the association between FPT and morbidity appears to vary across age-related risk groups, indicating that no single FPT cut-off is universally applicable. Therefore, contextual adjustments should be considered when adopting cut-offs or interpreting findings from published literature.

In agreement with Lombard et al. (20), the present calf-level study found a lower morbidity hazard in dairy calves born to primiparous compared to multiparous dams. However, this finding contrasts with other studies (19, 66) reporting a higher risk of morbidity in dairy calves from primiparous dams. Considering the greater FPT odds observed in calves from primiparous dams in the present calf-level study, a higher morbidity hazard in these calves was expected. This disparity suggests that the effect of dam parity on calf health is influenced by management practices and herd-specific factors other than passive immune status. Consistent with the present calf-level study, previous large-scale studies reported that dairy calves born with assistance (20) or sired by beef bulls (19) have a higher risk of experiencing morbidity events within the first 4 months of life, likely associated with FPT, as previously discussed. In the present calf-level study, over 80% of dairy calves were fed colostrum by hand, primarily using a nipple bottle or stomach tube. In contrast to the findings reported by Svensson et al. (19), this hand-feeding method was associated with an increased morbidity hazard compared to allowing calves to suckle “naturally” from their dams. One possible explanation for this finding may be poor hygiene of feeding equipment, which can lead to the build-up of disease-causing pathogens. For example, Renaud et al. (67) found that total bacterial and coliform counts in 59 and 21% of colostrum-feeding equipment tested on dairy farms, respectively, exceeded recommended levels.

The negative effect of housing beef cow-calf units in the same house as other cattle, as observed in the present herd-level study, concurs with a dairy study by Medrano-Galarza et al. (68), which reported increased within-pen prevalence of calf diarrhea and BRD when calf pens were located in barns shared with older cattle. This finding is likely due to increasing airborne pathogen density or direct pathogen transmission from older cattle to calves, which are more vulnerable to disease, within the housing environment (69). In accord with the present herd-level study, Trotz-Williams et al. (70) found that leaving dairy calves with the dams for more than 1 h post-birth was associated with increased risk for diarrhea. Although early cow-calf separation is a common practice on commercial dairy farms, its perceived benefits remain debatable. A review by Beaver et al. (71) found no scientific evidence that early cow-calf separation improves calf or cow health. Therefore, although the results of the present study suggest early separation may be beneficial, further research is necessary.

In the present herd-level study, the increased morbidity hazard in dairy calves on farms that did not clean pens between calf groups is likely due to the buildup of pathogens in organic matter (72, 73). This finding emphasizes the critical importance of thorough cleaning and disinfection of calf pens, including the complete removal of dirty bedding, to reduce pathogen transmission (72). Surprisingly, less than 20% of dairy farms in the herd-level study cleaned pens between calf groups. A more recent Irish study reported that 55% of dairy farms clean out calf pens, most apply disinfectant, on a fortnightly basis (74). While this suggests some improvement in hygiene practices on Irish dairy farms, it also highlights the need to encourage wider adoption of regular pen cleaning protocols.

### 4.4 Study limitations

First, the use of farmer-recorded morbidity data in the present study potentially introduces a risk of misclassification bias in the identification and categorization of morbidity events. Although standardized case definitions were provided to support morbidity recording (5), variability in farmer’s interpretation may have affected the data recording. Second, it should be acknowledged that the increasing population of dairy calves in Ireland in recent years (49) may have changed the herd dynamics or influenced the farm management practices, potentially affecting the current applicability of the findings. Despite these limitations, the study provides valuable insights, particularly given the limited existing data describing management practices associated with FPT and morbidity in Irish beef and dairy calves. Given the dynamic nature of herd demographics and evolving farm practices, ongoing research is essential to refine FPT benchmarks and inform effective interventions to improve calf immunity and health across production systems.

## 5 Conclusion

Passive immune tests and cut-off values substantially influence the interpretation of FPT and its associated risk factors in beef and dairy calves. In beef calves, the risk factors associated with passive immune measures and FPT included pre-calving dam diarrhea vaccination, dam parity, timing of birth in the calving season, calving area, breed, perinatal problems, and both the timing and method of colostrum feeding, and in dairy calves included length of dry period, dam parity, calving area, calving supervision, calf sex and breed, perinatal problems, and type of colostrum feeding.

In the conditions of the present study, passive immune measures were not significantly associated with morbidity in the first 30 days of life in beef and dairy calves. However, depending on passive immune tests and cut-off values, FPT was associated with increased morbidity hazard in beef, but not in dairy calves. The risk factors for morbidity in beef calves included housing location of the cow-calf units and dam parity. In dairy calves, timing of cow-calf separation, cleaning of pens between calf groups, dam parity, breed, calving assistance, and method of colostrum feeding were associated with morbidity. The findings can be used to inform targeted management strategies to enhance passive immunity and overall calf health, not only within Ireland but also in beef and dairy systems globally, particularly in regions with similar production systems.

## Data Availability

The data used in the present study are held by Teagasc and subjected to General Data Protection Regulation (GDPR). Research access to these data will be made available upon reasonable request to the corresponding author (bernadette.earley@teagasc.ie).
