# Correction: Factors associated with failure of passive transfer of immunity and morbidity in spring-born beef and dairy calves during the first 30 days of life

**DOI:** 10.3389/fvets.2026.1793243

**Published:** 2026-02-13

**Authors:** Rischi Robinson Male Here, Mark McGee, Catherine McAloon, Andrew W. Byrne, Bernadette Earley

**Affiliations:** 1 Animal and Grassland Research and Innovation Centre, Teagasc, Grange, Dunsany, Co. Meath, Ireland; 2 School of Veterinary Medicine, University College Dublin (UCD), Dublin, Ireland; 3 One Health Scientific Support Unit, Ruminant Animal Health Policy, Department of Agriculture, Food and the Marine (DAFM), Agriculture House, Dublin, Ireland

**Keywords:** calf, immunity tests, immunity cut-offs, calf management, disease susceptibility, risk factors, passive immunity

In the published article, there was a mistake in the **Results** section, and sentence “Beef calves born to primiparous dams had greater morbidity hazard than those born to multiparous dams.” was incorrect.

A correction has been made to section **3 Results**, **3.1 Herd-level study**, ***3.1.2 Morbidity***, Paragraph 2:

The correct sentence should read “Beef calves born to primiparous dams had lower morbidity hazard than those born to multiparous dams”.

The original version of this article has been updated.

