# Supplementary material for: Factors associated with failure of passive transfer of immunity and morbidity in spring-born beef and dairy calves during the first 30 days of life
Source: Front Vet Sci. 2026 Jan 6;12:1658532. doi: 10.3389/fvets.2025.1658532 (PMC12815765; doi:10.3389/fvets.2025.1658532)
Supplement: Supplementary file 1 [file Table_1.docx]

# **Supplementary Materials**

**Supplementary Table 1.** Pearson’s correlation coefficient between passive immune measures, and Cohen’s Kappa agreement between failure of passive transfer of immunity (FPT), defined by current-cut-offs and literature-cut-offs of ELISA-IgG, TP-CA, and TS-BRIX, in beef and dairy calves.

| **Testing methods** | **Pearson's Correlation** | |  | **Cohen's Kappa (CI 95%)^1^** | | | | | |
| --- | --- | --- | --- | --- | --- | --- | --- | --- | --- |
|  | **TP-CA** | **TS-BRIX** |  | **CC *vs.* LC** | **CC^2^** | |  | **LC^3^** | |
|  |  |  |  |  | **TP-CA** | **TS-BRIX** |  | **TP-CA** | **TS-BRIX** |
| **Herd-level study** |  |  |  |  |  |  |  | |  |
| **Beef** | | |  |  |  |  |  | |  |
| ELISA-IgG | 0.75 | 0.72 |  | 0.84 (0.78 - 0.90) | 0.53 (0.45 - 0.61) | 0.54 (0.45 - 0.63) | 0.40 (0.31 - 0.49) | | 0.57 (0.48 - 0.66) |
| TP-CA | – | 0.91 |  | 0.33 (0.26 - 0.40) | – | 0.71 (0.64 - 0.78) | – | | 0.52 (0.43 - 0.61) |
| TS-BRIX | – | – |  | 0.89 (0.84 - 0.94) | – | – | – | | – |
| **Dairy** | | |  |  |  |  |  | |  |
| ELISA-IgG | 0.79 | 0.76 |  | 0.64 (0.58 - 0.71) | 0.62 (0.57 - 0.70) | 0.58 (0.51 - 0.64) | 0.55 (0.46 - 0.64) | | 0.63 (0.55 - 0.70) |
| TP-CA | – | 0.92 |  | 0.34 (0.27 - 0.40) | – | 0.71 (0.65 - 0.77) | – | | 0.52 (0.43 - 0.60) |
| TS-BRIX | – | – |  | 0.84 (0.80 - 0.89) | – | – | – | | – |
| **Calf-level study** |  |  |  |  |  |  |  | |  |
| **Beef** |  |  |  |  |  |  |  | |  |
| ELISA-IgG | 0.86 | 0.80 |  | 0.92 (0.87 - 0.96) | 0.44 (0.36 - 0.52) | 0.57 (0.48 - 0.66) | 0.66 (0.57 - 0.75) | | 0.66 (0.57 - 0.74) |
| TP-CA | – | 0.95 |  | 0.35 (0.27 - 0.42) | – | 0.67 (0.61 - 0.75) | – | | 0.70 (0.61 - 0.78) |
| TS-BRIX | – | – |  | 0.85 (0.79 - 0.91) | – | – | – | | – |
| **Dairy** | | |  |  |  |  |  | |  |
| ELISA-IgG | 0.85 | 0.80 |  | 0.73 (0.68 - 0.78) | 0.69 (0.64 - 0.74) | 0.62 (0.56 - 0.67) | 0.56 (0.50 - 0.64) | | 0.65 (0.59 - 0.71) |
| TP-CA | – | 0.95 |  | 0.32 (0.27 - 0.38) | – | 0.73 (0.68 - 0.77) | – | | 0.57 (0.48 - 0.62) |
| TS-BRIX | – | – |  | 0.83 (0.79 - 0.87) | – | – | – | | – |

^1^Cohen’s Kappa agreement: Fair (0.20-0.40), Moderate (0.41- 0.60), Substantial (0.61-0.80), Almost perfect (0.81-1.00); ^2^CC – FPT defined by current-cut-offs (ELISA-IgG ≤9 mg/mL, TP-CA ≤60 g/L, TS-BRIX ≤8.4% for beef; ELISA-IgG ≤12 mg/mL, TP-CA ≤60 g/L, TS-BRIX ≤8.4% for dairy calves); ^3^LC – FPT defined by literature-cut-offs (ELISA-IgG <10 mg/mL, TP-CA <52 g/L, TS-BRIX <8.4% for both beef and dairy calves).

**Supplementary Table 2.** Performance of current-cut-offs and literature-cut-offs of TP-CA and TS-BRIX in identifying failure of passive transfer of immunity (FPT), defined by both current-cut-off and literature-cut-off of ELISA-IgG as the “standard” references, in beef and dairy calves.

| **Tests performance** | **Cut-off values** | **CC^1^ of ELISA-IgG as standard reference** | | | | |  | **LC^2^ of ELISA-IgG as standard reference** | | | | |
| --- | --- | --- | --- | --- | --- | --- | --- | --- | --- | --- | --- | --- |
|  |  | **Sensitivity** | **Specificity** | **PPV** | **NPV** | **Accuracy** |  | **Sensitivity** | **Specificity** | **PPV** | **NPV** | **Accuracy** |
| **Herd-level study** |  |  |  |  |  |  |  |  |  |  |  |  |
| **Beef** |  |  |  |  |  |  |  |  |  |  |  |  |
| TP-CA (CC) | ≤60 g/L | 0.89 | 0.73 | 0.56 | 0.95 | 0.78 |  | 0.87 | 0.79 | 0.69 | 0.92 | 0.82 |
| TP-CA (LC) | <52 g/L | 0.44 | 0.98 | 0.90 | 0.82 | 0.83 |  | 0.36 | 0.98 | 0.92 | 0.74 | 0.77 |
| TS-BRIX (CC) | ≤8.4% | 0.78 | 0.81 | 0.61 | 0.91 | 0.80 |  | 0.73 | 0.85 | 0.72 | 0.86 | 0.81 |
| TS-BRIX (LC) | <8.4% | 0.73 | 0.86 | 0.66 | 0.89 | 0.82 |  | 0.67 | 0.89 | 0.76 | 0.83 | 0.81 |
| **Dairy** |  |  |  |  |  |  |  |  |  |  |  |  |
| TP-CA (CC) | ≤60 g/L | 0.76 | 0.88 | 0.73 | 0.89 | 0.84 |  | 0.91 | 0.81 | 0.50 | 0.98 | 0.83 |
| TP-CA (LC) | <52 g/L | 0.27 | 1.00 | 0.96 | 0.76 | 0.78 |  | 0.45 | 0.99 | 0.91 | 0.90 | 0.90 |
| TS-BRIX (CC) | ≤8.4% | 0.64 | 0.91 | 0.75 | 0.86 | 0.83 |  | 0.82 | 0.86 | 0.54 | 0.96 | 0.85 |
| TS-BRIX (LC) | <8.4% | 0.55 | 0.95 | 0.82 | 0.83 | 0.83 |  | 0.76 | 0.91 | 0.64 | 0.95 | 0.89 |
| **Calf-level study** |  |  |  |  |  |  |  |  |  |  |  |  |
| **Beef** |  |  |  |  |  |  |  |  |  |  |  |  |
| TP-CA (CC) | ≤60 g/L | 0.96 | 0.65 | 0.46 | 0.98 | 0.72 |  | 0.96 | 0.68 | 0.53 | 0.98 | 0.75 |
| TP-CA (LC) | <52 g/L | 0.66 | 0.98 | 0.91 | 0.90 | 0.90 |  | 0.60 | 0.99 | 0.94 | 0.87 | 0.88 |
| TS-BRIX (CC) | ≤8.4% | 0.85 | 0.81 | 0.59 | 0.94 | 0.82 |  | 0.83 | 0.83 | 0.65 | 0.93 | 0.83 |
| TS-BRIX (LC) | <8.4% | 0.81 | 0.88 | 0.69 | 0.94 | 0.87 |  | 0.77 | 0.90 | 0.74 | 0.91 | 0.86 |
| **Dairy** |  |  |  |  |  |  |  |  |  |  |  |  |
| TP-CA (CC) | ≤60 g/L | 0.85 | 0.86 | 0.74 | 0.93 | 0.86 |  | 0.93 | 0.78 | 0.54 | 0.98 | 0.81 |
| TP-CA (LC) | <52 g/L | 0.32 | 1.00 | 1.00 | 0.76 | 0.78 |  | 0.46 | 1.00 | 0.97 | 0.87 | 0.88 |
| TS-BRIX (CC) | ≤8.4% | 0.68 | 0.91 | 0.79 | 0.86 | 0.84 |  | 0.81 | 0.87 | 0.62 | 0.94 | 0.86 |
| TS-BRIX (LC) | <8.4% | 0.59 | 0.96 | 0.88 | 0.83 | 0.84 |  | 0.73 | 0.93 | 0.72 | 0.93 | 0.88 |

PPV – Positive predictive value; NPV – Negative predictive value; ^1^CC – FPT defined by current-cut-offs (ELISA-IgG ≤9 mg/mL, TP-CA ≤60 g/L, TS-BRIX ≤8.4% for beef; ELISA-IgG ≤12 mg/mL, TP-CA ≤60 g/L, TS-BRIX ≤8.4% for dairy calves); ^2^LC – FPT defined by literature-cut-offs (ELISA-IgG <10 mg/mL, TP-CA <52 g/L, TS-BRIX <8.4% for both beef and dairy calves).

**Supplementary Table 3.** Results from mixed multivariable linear regression models identifying factors associated with passive immune measures in beef calves, evaluated using ELISA-IgG, TP-CA, and TS-BRIX, in the herd-level study.

| **Variable** | **Category** | **ELISA-IgG^1^ (*n* = 391)** | | | | | **TP-CA (*n* = 391)** | | | | | | | **TS-BRIX (*n* = 391)** | | | | | |
| --- | --- | --- | --- | --- | --- | --- | --- | --- | --- | --- | --- | --- | --- | --- | --- | --- | --- | --- | --- |
|  |  | **β** | **CI 95%** | | ***P*-value** | | **β** | | | **CI 95%** | | ***P*-value** | | **β** | | **CI 95%** | | ***P*-value** | |
| *Intercept* |  | 3.95 | 3.71 to 4.17 | | <0.0001 | | 63.91 | | | 61.87 to 65.95 | | <0.0001 | | 9.31 | | 9.03 to 9.60 | | <0.0001 | |
| *Dam diarrhoea vaccination pre-calving* | Yes | Ref. | Ref. | | Ref. | |  | | | NS | |  | | Ref. | | Ref. | | Ref. | |
|  | No | -0.27 | -0.48 to -0.05 | | 0.02 | |  | | |  |  |  | | -0.31 | | -0.59 to -0.02 | | 0.03 | |
| *Age at sampling (day)* | Mean 8, SD 4, median 8 | -0.05 | -0.07 to -0.03 | | <0.0001 | | -0.33 | | | -0.54 to -0.13 | | 0.002 | | -0.04 | | -0.06 to -0.02 | | 0.001 | |
| *Farm-level variance (ICC)^†^* |  | 0.19 | |  | |  | | 0.19 |  | |  | | 0.23 | |  | |  | |  |

NS – Not significant; **^†^**ICC – Intra-class correlation; ^1^Square root transformed

**Supplementary Table 4.** Results from mixed multivariable logistic regression models identifying factors associated with failure of passive transfer of immunity (FPT) in beef calves, defined by current-cut-offs and literature-cut-offs of ELISA-IgG, TP-CA, TS-BRIX, in the herd-level study.

| **Variable** | **Category** | **ELISA-IgG** | | **TP-CA** | | **TS-BRIX** | |
| --- | --- | --- | --- | --- | --- | --- | --- |
|  |  | **OR (CI 95%)** | ***P*-value** | **OR (CI 95%)** | ***P*-value** | **OR (CI 95%)** | ***P*-value** |
| **Current-cut-offs** |  | ***n* = 391** |  | ***n* = 391** |  | ***n* = 391** |  |
| *Dam diarrhoea vaccination pre-calving* | Yes | Ref. | Ref. | Ref. | Ref. | Ref. | Ref. |
|  | No | 1.87 (1.04 - 3.37) | 0.04 | 1.70 (1.01 - 2.87) | 0.05 | 1.97 (1.02 - 3.79) | 0.04 |
| *Age at sampling (day)* | Mean 8, SD 4, median 8 | 1.14 (1.07- 1.22) | 0.0001 | 1.08 (1.02 - 1.14) | 0.01 | 1.07 (1.00 - 1.14) | 0.05 |
| *Farm-level variance (ICC)^†^* |  | 0.12 |  | 0.11 |  | 0.22 |  |
|  |  |  |  |  |  |  |  |
| **Literature-cut-offs** |  | ***n* = 391** |  | ***n* = 391** |  | ***n* = 391** |  |
| *Dam diarrhoea vaccination pre-calving* | Yes | Ref. | Ref. | NS |  | NS |  |
|  | No | 1.92 (1.10 - 3.35) | 0.02 |  |  |  |  |
| *Age at sampling (day)* | Mean 8, SD 4, median 8 | 1.15 (1.08 - 1.22) | <0.0001 | 1.01 (0.93 - 1.09) | 0.88 | 1.04 (0.97 - 1.11) | 0.24 |
| *Farm-level variance (ICC)^†^* |  | 0.12 |  | 0.24 |  | 0.20 |  |

NS – Not significant; **^†^**ICC – Intra-class correlation

**Supplementary Table 5.** Results from mixed multivariable linear regression models identifying factors associated with passive immune measures in dairy calves, evaluated using ELISA-IgG, TP-CA, and TS-BRIX, in the herd-level study.

| **Variable** | **Category** | **ELISA-IgG^1^ (*n* = 674)** | | | **TP-CA (*n* = 668)** | | | **TS-BRIX (*n* = 668)** | | |
| --- | --- | --- | --- | --- | --- | --- | --- | --- | --- | --- |
|  |  | **β** | **CI 95%** | ***P*-value** | **β** | **CI 95%** | ***P*-value** | **β** | **CI 95%** | ***P*-value** |
| *Intercept* |  | 4.28 | 4.12 to 4.43 | <0.0001 | 68.98 | 67.23 to 70.74 | <0.0001 | 9.65 | 9.46 to 9.84 | <0.0001 |
| *Dam parity* | Multiparous |  | NS |  | Ref. | Ref. | Ref. | Ref. | Ref. | Ref. |
|  | Primiparous |  |  |  | -1.61 | -3.01 to -0.20 | 0.03 | -0.16 | -0.32 to -0.01 | 0.04 |
| *Calf sex* | Female |  | NS |  | Ref. | Ref. | Ref. |  | NS |  |
|  | Male |  |  |  | -1.60 | -2.88 to -0.31 | 0.02 |  |  |  |
| *Age at sampling (day)* | Mean 7, SD 4, median 7 | -0.07 | -0.08 to -0.05 | <0.0001 | -0.53 | -0.71 to -0.35 | <0.0001 | -0.07 | -0.09 to -0.05 | <0.0001 |
| *Farm-level variance (ICC)^†^* |  | 0.15 |  |  | 0.14 |  |  | 0.17 |  |  |

NS – Not significant; **^†^**ICC – Intra-class correlation; ^1^Square root transformed

**Supplementary Table 6.** Results from mixed multivariable logistic regression models identifying factors associated with failure of passive transfer of immunity (FPT) in dairy calves, defined by current-cut-offs and literature-cut-offs of ELISA-IgG, TP-CA, TS-BRIX, in the herd-level study.

| **Variable** | **Category** | **ELISA-IgG** | | **TP-CA** | | **TS-BRIX** | |
| --- | --- | --- | --- | --- | --- | --- | --- |
|  |  | **OR (CI 95%)** | ***P*-value** | **OR (CI 95%)** | ***P*-value** | **OR (CI 95%)** | ***P*-value** |
| **Current-cut-offs** |  | ***n* = 674** | | ***n* = 668** | | ***n* = 668** | |
| *Dam parity* | Multiparous | NS |  | Ref. | Ref. | Ref. | Ref. |
|  | Primiparous |  |  | 1.66 (1.10 - 2.59) | 0.02 | 1.95 (1.27 - 2.99) | 0.002 |
| *Calf sex* | Female | Ref. | Ref. | Ref. | Ref. | NS |  |
|  | Male | 1.48 (1.00 - 2.19) | 0.05 | 1.67 (1.14 - 2.44) | 0.01 |  |  |
| *Length of dry period* | >8 weeks | Ref. | Ref. | Ref. | Ref. | Ref. | Ref. |
|  | ≤8 weeks | 1.79 (1.03 - 3.12) | 0.03 | 1.74 (1.03 - 2.96) | 0.04 | 1.81 (1.04 - 3.15) | 0.04 |
| *Age at sampling (day)* | Mean 7, SD 4, median 7 | 1.17 (1.10 - 1.23) | <0.0001 | 1.10 (1.04 - 1.16) | 0.0006 | 1.11 (1.05 - 1.17) | 0.0005 |
| *Farm-level variance (ICC)^†^* |  | 0.18 |  | 0.15 |  | 0.16 |  |
|  |  |  |  |  |  |  |  |
| **Literature-cut-offs** |  | ***n* = 674** |  | ***n* = 674** |  | ***n* = 674** |  |
| *Calf sex* | Female | Ref. | Ref. | Ref. | Ref. | NS |  |
|  | Male | 1.63 (1.04 – 2.57) | 0.04 | 2.39 (1.31 - 4.38) | 0.01 |  |  |
| *Length of dry period* | >8 weeks | Ref. | Ref. | Ref. | Ref. | NS |  |
|  | ≤8 weeks | 2.01 (1.11 - 3.62) | 0.02 | 2.10 (1.00 - 4.42) | 0.05 |  |  |
| *Age at sampling (day)* | Mean 7, SD 4, median 7 | 1.13 (1.06 - 1.21) | 0.001 | 1.09 (0.99 - 1.18) | 0.07 | 1.12 (1.06 - 1.19) | 0.0002 |
| *Farm-level variance (ICC)^†^* |  | 0.16 |  | 0.20 |  | 0.17 |  |

NS – Not significant; **^†^**ICC – Intra-class correlation.

**Supplementary Table 7.** Calf-level colostrum management in beef and dairy calves, and between male and female dairy calves in the calf-level study.

| **Calf-level colostrum management^1, 2^** | **Beef (*n* = 377)** | | **Dairy (*n* = 916)** | | ***P*-value** | **Dairy^3^** | | | | ***P*-value** |
| --- | --- | --- | --- | --- | --- | --- | --- | --- | --- | --- |
|  |  |  |  |  |  | **Female (*n* = 479)** | | **Male (*n* = 435)** | |  |
|  | ***n*** | **%** | ***n*** | **%** |  | ***n*** | **%** | ***n*** | **%** |  |
| *Time of first colostrum feeding after birth* |  |  |  |  |  |  |  |  |  |  |
| >2 h | 41 | 19.5 | 154 | 22.8 | 0.315 | 86 | 23.8 | 68 | 21.7 | 0.503 |
| Within 2 h | 169 | 80.5 | 521 | 77.2 |  | 275 | 76.2 | 246 | 78.3 |  |
| *Method of colostrum feeding* |  |  |  |  |  |  |  |  |  |  |
| Suckled dam without assistance | 276 | 73.2 | 168 | 18.5 | <0.0001 | 83 | 17.4 | 83 | 19.3 | 0.458 |
| Suckled dam with assistance | 76 | 20.2 | 2 | 0.2 |  | 1 | 0.2 | 1 | 0.2 |  |
| Hand-fed | 25 | 6.6 | 739 | 81.3 |  | 393 | 82.4 | 346 | 80.5 |  |
| *Method of colostrum feeding if hand-fed* |  |  |  |  |  |  |  |  |  |  |
| Bucket | – | – | 76 | 10.3 | <0.0001 | 36 | 9.2 | 9 | 2.6 | 0.653 |
| Bucket with teat | – | – | 23 | 3.1 |  | 14 | 3.6 | 40 | 11.6 |  |
| Nipple bottle | 6 | 24.0 | 412 | 55.9 |  | 224 | 57.1 | 188 | 54.5 |  |
| Stomach tube | 19 | 76.0 | 226 | 30.7 |  | 118 | 30.1 | 108 | 31.3 |  |
| *Volume of first colostrum feeding* |  |  |  |  |  |  |  |  |  |  |
| 3 L or More | – | – | 459 | 53.6 | <0.0001 | 240 | 53.3 | 219 | 54.2 | 0.538 |
| <3 L | 25 | 6.6 | 227 | 26.5 |  | 126 | 28.0 | 101 | 25.0 |  |
| Suckled directly from dam | 352 | 93.4 | 170 | 19.9 |  | 84 | 18.7 | 84 | 20.8 |  |
| *Volume of first colostrum feeding if hand-fed* | 1.5 to 2 L  (mean 2, SD 0.1, median 2 L, *n = 25*) | | 0.5 to 4.5 L  (mean 2.8, SD 0.4, median 3 L, *n* = 686) | |  | 1 to 4.5 L  (mean 2.8, SD 0.4, median 3 L, *n =* 366) | | 0.5 to 4.5 L  (mean 2.9, SD 0.5, median 3 L, *n =* 320) | |  |
| *Total volume of colostrum in the first 24 hours* |  |  |  |  |  |  |  |  |  |  |
| 6 L or more | – | – | 265 | 31.5 | <0.0001 | 146 | 33.2 | 119 | 29.8 | 0.540 |
| <6 L | 15 | 4.1 | 406 | 48.3 |  | 210 | 47.7 | 196 | 49.1 |  |
| Suckled directly from dam | 352 | 95.9 | 170 | 20.2 |  | 84 | 19.1 | 84 | 21.1 |  |
| *Total volume of colostrum in the first 24 hours if hand-fed* | 2 to 4 L  (mean 2.1, SD 0.5, median 2 L, *n =* 15) | | 2 to 12 L  (mean 5.4, SD 1.5, median 5 L, *n* = 671) | |  | 2 to 12 L  (mean 5.5, SD 1.6, median 5 L, *n =* 356) | | 2.5 to 10 L  (mean 5.3, SD 1.3, median 5 L, *n* = 315) | |  |
| *Source of colostrum* |  |  |  |  |  |  |  |  |  |  |
| From dam only^4^ | 359 | 95.2 | 575 | 63.3 | <0.0001 | 302 | 63.3 | 271 | 63.0 | 0.928 |
| From donor cow/cows | – | – | 334 | 36.7 |  | 175 | 36.7 | 159 | 37.0 |  |
| Artificial colostrum | 4 | 1.1 | – | – |  | – | – | – | – |  |
| Obtained from another herd | 14 | 3.7 | – | – |  | – | – | – | – |  |
| *Type of colostrum* |  |  |  |  |  |  |  |  |  |  |
| Freshly harvested^5^ | 12 | 3.2 | 704 | 77.5 | <0.0001 | 374 | 78.4 | 330 | 76.7 | 0.751 |
| Stored colostrum | 13 | 3.5 | 35 | 3.9 |  | 19 | 4.0 | 16 | 3.7 |  |
| Suckled directly from dam | 352 | 93.4 | 170 | 18.7 |  | 84 | 17.6 | 84 | 19.5 |  |

^1^Sums for each variable varied due to missing data; ^2^Rows with counts less than 5 were excluded from the Chi-square (χ^2^) analysis; ^3^Sex data was missing for two dairy calves; ^4^Harvested colostrum or suckled directly from dam; ^5^Including artificial colostrum.

**Supplementary Table 8.** Results from mixed multivariable linear regression models identifying factors associated with passive immune measures in beef calves, evaluated using ELISA-IgG, TP-CA, and TS-BRIX, in the calf-level study.

| **Variable** | **Category** | **ELISA-IgG^1^ (*n* = 210)** | | | **TP-CA (*n* = 210)** | | | | **TS-BRIX (*n* = 210)** | | |  |
| --- | --- | --- | --- | --- | --- | --- | --- | --- | --- | --- | --- | --- |
|  |  | **β** | **CI 95%** | ***P*-value** | | **β** | **CI 95%** | ***P*-value** | **β** | **CI 95%** | ***P*-value** | |
| *Intercept* |  | 3.89 | 3.43 to 4.35 | <0.0001 | | 61.11 | 56.81 to 65.41 | <0.0001 | 8.87 | 8.40 to 9.35 | <0.0001 | |
| *Timing of birth in the calving season* | Early | Ref. | Ref. | Ref. | |  |  |  |  |  |  | |
|  | Peak | -0.31 | -0.56 to -0.06 | 0.02 | |  | NS |  |  | NS |  | |
|  | Late | 0.25 | -0.06 to 0.56 | 0.12 | |  |  |  |  |  |  | |
| *Perinatal problems^2^* | No | Ref. | Ref. | Ref. | | Ref. | Ref. | Ref. | Ref. | Ref. | Ref. | |
|  | Yes | -0.44 | -0.78 to -0.11 | 0.01 | | -6.58 | -9.85 to -3.32 | <0.0001 | -0.69 | -1.06 to -0.32 | 0.0003 | |
| *Time of first colostrum feeding post-birth* | Within 2 h | Ref. | Ref. | Ref. | | Ref. | Ref. | Ref. | Ref. | Ref. | Ref. | |
|  | >2 h | -0.43 | -0.71 to -0.15 | 0.003 | | -3.29 | -6.02 to -0.57 | 0.02 | -0.42 | 0.73 to -0.12 | 0.01 | |
| *Age at sampling (day)* | Mean 8, SD 4, median 8 | -0.03 | -0.06 to 0.003 | 0.08 | | -0.02 | -0.32 to 0.27 | 0.87 | 0.01 | -0.03 to 0.04 | 0.74 | |
| *Farm-level variance (ICC)^†^* |  | 0.25 |  |  | | 0.25 |  |  | 0.23 |  |  | |

NS – Not significant; **^†^**ICC – Intra-class correlation; ^1^Square root transformed; ^2^Perinatal problems included: weak, no-suckle responses, dullness, being reluctant to stand, standing difficulty, or miss-mothering.

**Supplementary Table 9.** Results from mixed multivariable logistic regression models identifying factors associated with failure of passive transfer of immunity (FPT) in beef calves, defined by current-cut-offs and literature cut-offs of ELISA-IgG, TP-CA, and TS-BRIX, in the calf-level study.

| **Variable** | **Category** | **ELISA-IgG** | | **TP-CA** | | **TS-BRIX** | |
| --- | --- | --- | --- | --- | --- | --- | --- |
|  |  | **OR (CI 95%)** | ***P*-value** | **OR (CI 95%)** | ***P*-value** | **OR (CI 95%)** | ***P*-value** |
| **Current-cut-offs** | | ***n* = 210** |  | ***n* = 210** |  | ***n* = 377** |  |
| *Dam parity* | Multiparous | NS |  | Ref. | Ref. | NS |  |
|  | Primiparous |  |  | 3.10 (1.28 - 7.52) | 0.01 |  |  |
| *Timing of birth in the calving season* | Early | Ref. | Ref. |  |  |  |  |
|  | Peak | 3.84 (1.56 - 9.44) | 0.004 | NS |  | NS |  |
|  | Late | 0.26 (0.08 - 0.87) | 0.03 |  |  |  |  |
| *Breed* | Early maturing | Ref. | Ref. | NS |  | NS |  |
|  | Late maturing | 3.17 (1.03 - 9.75) | 0.04 |  |  |  |  |
| *Calving area* | Individual calving pen |  |  | Ref. | Ref. |  |  |
|  | Group calving pen | NS |  | 0.28 (0.11 - 0.71) | 0.01 | NS |  |
|  | Other^1^ |  |  | 0.70 (0.15 - 3.26) | 0.65 |  |  |
| *Perinatal problems* | No | Ref. | Ref. | Ref. | Ref. | Ref. | Ref. |
|  | Yes^2^ | 4.27 (1.38 - 13.19) | 0.01 | 3.68 (1.18 - 11.50) | 0.03 | 4.07 (1.69 - 9.79) | 0.002 |
| *Time of first colostrum feeding post-birth* | Within 2 h | Ref. | Ref. | Ref. | Ref. | NS |  |
|  | >2 h | 10.12 (3.33 - 30.83) | <0.0001 | 5.79 (2.11 - 15.92) | 0.001 |  |  |
| *Age at sampling (day)* | Mean 8, SD 4, median 8 | 1.10 (0.99 - 1.22) | 0.08 | 0.98 (0.89 - 1.08) | 0.68 | 1.00 (0.94 - 1.07) | 0.99 |
| *Farm-level variance (ICC)^†^* |  | 0.56 |  | 0.28 |  | 0.22 |  |
|  |  |  |  |  |  |  |  |
| **Literature-cut-offs** | | ***n* = 210** |  | ***n* = 210** |  | ***n* = 210** |  |
| *Time of birth relative to calving season* | Early | Ref. | Ref. |  |  |  |  |
|  | Peak | 2.52 (1.09 - 5.81) | 0.03 | NS |  | NS |  |
|  | Late | 0.35 (0.12 - 1.04) | 0.06 |  |  |  |  |
| *Perinatal problems* | No | Ref. | Ref. | Ref. | Ref. | Ref. | Ref. |
|  | Yes | 4.53 (1.50 - 13.70) | 0.01 | 4.12 (1.27 - 13.34) | 0.02 | 3.63 (1.41 - 9.33) | 0.01 |
| *Time of first colostrum feeding post-birth* | Within 2 h | Ref. | Ref. | Ref. | Ref. | Ref. | Ref. |
|  | >2 h | 5.12 (1.91 - 14.03) | 0.001 | 2.89 (1.18 - 7.07) | 0.02 | 2.34 (1.02 - 4.93) | 0.05 |
| *Method of colostrum feeding* | Suckled dam without assistance |  |  | Ref. | Ref. |  |  |
|  | Suckled dam with assistance | NS |  | 2.99 (1.18 - 7.60) | 0.02 | NS |  |
|  | Hand-fed |  |  | 0.64 (0.11 - 3.91) | 0.63 |  |  |
| *Age at sampling (day)* | Mean 8, SD 4, median 8 | 1.11 (1.00 - 1.23) | 0.04 | 1.01 (0.91 - 1.12) | 0.86 | 1.00 (0.92 - 1.09) | 0.96 |
| *Farm-level variance (ICC)^†^* |  | 0.50 |  | 0.26 |  | 0.17 |  |

NS – Not significant; **^†^**ICC – Intra-class correlation; ^1^Other calving area included: outdoors, slats, cubicles, dry cow pens, handling crush; ^2^Perinatal problems included: weak, no-suckle responses, dullness, being reluctant to stand, standing difficulty, or miss-mothering.

**Supplementary Table 10.** Results from mixed multivariable linear regression models identifying factors associated with passive immune measures in dairy calves, evaluated using ELISA-IgG, TP-CA, and TS-BRIX, in the calf-level study.

| **Variables** | **Category** | **ELISA-IgG (*n* = 914)** | | | **TP-CA (*n* = 910)** | | | **TS-BRIX (*n* = 914)** | | |
| --- | --- | --- | --- | --- | --- | --- | --- | --- | --- | --- |
|  |  | **β** | **CI 95%** | ***P*-value** | **β** | **CI 95%** | ***P*-value** | **β** | **CI 95%** | ***P*-value** |
| *Intercept* |  | 16.40 | 15.48 to 17.32 | <0.0001 | 65.04 | 63.26 to 66.81 | <0.0001 | 9.35 | 9.11 to 9.58 | <0.0001 |
| *Dam parity* | Multiparous | Ref. | Ref. | Ref. | Ref. | Ref. | Ref. | Ref. | Ref. | Ref. |
|  | Primiparous | -1.56 | -2.30 to -0.81 | <0.0001 | -2.51 | -3.67 to -1.35 | <0.0001 | -0.24 | -0.37 to -0.10 | 0.001 |
| *Perinatal problems^1^* | No |  | NS |  | Ref. | Ref. | Ref. |  | NS |  |
|  | Yes |  |  |  | -3.79 | -7.43 to -0.15 | 0.041 |  |  |  |
| *Age at sampling (day)* | Mean 8, SD 4, median 8 | -0.25 | -0.33 to -0.16 | <0.0001 | -0.22 | -0.35 to -0.09 | 0.001 | -0.03 | -0.04 to -0.01 | 0.0002 |
| *Farm-level variance (ICC)^†^* |  | 0.02 |  |  | 0.04 |  |  | 0.06 |  |  |

NS – Not significant; **^†^**ICC – Intra-class correlation; ^1^Perinatal problems included: weak, no-suckle responses, dullness, being reluctant to stand, standing difficulty, or miss-mothering.

**Supplementary Table 11.** Results from mixed multivariable logistic regression models identifying factors associated with failure of passive transfer of immunity (FPT) in dairy calves, defined by current-cut-offs and literature cut-offs of ELISA-IgG, TP-CA, and TS-BRIX, in the calf-level study.

| **Variable** | **Category** | **ELISA-IgG** | | **TP-CA** | | **TS-BRIX** | |
| --- | --- | --- | --- | --- | --- | --- | --- |
|  |  | **OR (CI 95%)** | ***P*-value** | **OR (CI 95%)** | ***P*-value** | **OR (CI 95%)** | ***P*-value** |
| **Current-cut-offs** |  | ***n* = 909** | | ***n* = 909** | | ***n* = 909** | |
| *Dam parity* | Multiparous | Ref. | Ref. | Ref. | Ref. | NS |  |
|  | Primiparous | 1.59 (1.16 - 2.18) | 0.004 | 1.50 (1.10 - 2.04) | 0.01 |  |  |
| *Breed* | Dairy-sired | NS |  | Ref. | Ref. | Ref. | Ref. |
|  | Beef-sired (dairy-beef) |  |  | 1.55 (1.09 - 2.21) | 0.02 | 1.47 (1.01 - 2.13) | 0.05 |
| *Calving supervision* | Someone presents for calving | Ref. | Ref. | Ref. | Ref. | Ref. | Ref. |
|  | Calving not supervised | 0.71 (0.53 - 0.97) | 0.03 | 0.63 (0.46 - 0.85) | 0.002 | 0.59 (0.42 - 0.81) | 0.001 |
| *Age at sampling (day)* | Mean 8, SD 4, median 8 | 1.06 (1.02 - 1.10) | 0.002 | 1.05 (1.01 - 1.08) | 0.02 | 1.02 (0.98 - 1.06) | 0.45 |
| *Farm-level variance (ICC)^†^* |  | 0.03 |  | 0.04 |  | 0.07 |  |
|  |  |  |  |  |  |  |  |
| **Literature-cut-offs** |  | ***n* = 909** | | ***n* = 906** | | ***n* = 907** | |
| *Dam parity* | Multiparous | Ref. | Ref. | NS |  | NS |  |
|  | Primiparous | 1.55 (1.09 - 2.20) | 0.02 |  |  |  |  |
| *Calving area* | Individual calving pen |  |  | Ref. | Ref. |  |  |
|  | Group calving pen | NS |  | 6.71 (1.89 - 23.85) | 0.003 | NS |  |
|  | Other^1^ |  |  | 9.05 (2.19 - 37.45) | 0.002 |  |  |
| *Calving supervision* | Someone presents for calving | Ref. | Ref. | Ref. | Ref. | Ref. | Ref. |
|  | Calving not supervised | 0.61 (0.43 - 0.87) | 0.01 | 0.61 (0.37 - 0.99) | 0.05 | 0.58 (0.40 - 0.83) | 0.003 |
| *Type of colostrum feeding* | Freshly harvested |  |  | Ref. | Ref. | Ref. | Ref. |
|  | Stored colostrum | NS |  | 5.02 (1.54 - 16.35) | 0.01 | 2.76 (1.22 - 6.22) | 0.02 |
|  | Suckled direct from dam |  |  | 0.86 (0.47 - 1.56) | 0.61 | 1.19 (0.76 - 1.88) | 0.45 |
| *Age at sampling (day)* | Mean 8, SD 4, median 8 | 1.04 (1.00 - 1.08) | 0.08 | 0.97 (0.92 - 1.03) | 0.34 | 1.02 (0.98 - 1.07) | 0.33 |
| *Farm-level variance (ICC)^†^* |  | 0.07 |  | 0.10 |  | 0.12 |  |

NS – Not significant; **^†^**ICC – Intra-class correlation; ^1^Other calving area included: outdoors, slats, cubicles, dry cow pens, handling crush.
